# Generation of a spontaneous murine HPV + oral cancer model with site-specific oncogene insertion using CRISPR-SONIC

**DOI:** 10.1186/s13578-025-01427-5

**Published:** 2025-06-18

**Authors:** Julia Tao, Jason Murray, Hsin-Fang Tu, Darrell Fan, Ya-Chea Tsai, Ming-Hung Hu, Annie A. Wu, Deyin Xing, Chien-Fu Hung, T.-C. Wu

**Affiliations:** 1 Department of Pathology, Johns Hopkins University School of Medicine, 1550 Orleans Street, CRB II 307, Baltimore, MD 21287 USA; 2 Department of Surgery, Yale School of Medicine, Yale University, New Haven, CT USA; 3 Division of Hematology and Oncology, Department of Internal Medicine, Wan Fang Hospital, Taipei Medical University, Taipei, Taiwan; 4 Department of Oncology, Johns Hopkins School of Medicine, Baltimore, MD USA; 5 Department of Obstetrics and Gynecology, Johns Hopkins School of Medicine, Baltimore, MD USA; 6 Department of Molecular Microbiology and Immunology, Bloomberg School of Public Health, Johns Hopkins School of Medicine, Baltimore, MD USA; 7 The Johns Hopkins University School of Medicine, 1550 Orleans St, CRB II Room 309, Baltimore, MD 21287 USA

**Keywords:** Head and neck cancer, Murine model, Human papillomavirus, HPV16, E6, E7, Ras, CRISPR-SONIC, Buccal tumor, Immunotherapy

## Abstract

Human papillomavirus associated head and neck cancer (HPV + HNC) is rising globally, emphasizing the need for improved therapeutic and screening strategies. To test novel therapies and study HPV-related disease progression, it is vital to develop relevant preclinical models. However, many fail to address critical concerns, including generating a representative immune microenvironment and adequately modeling HPV-driven malignant transformation. Recent multi-omics studies reveal the significance of HPV integration location in HPV-related carcinogenesis and highlight the necessity of targeted treatment methods. Thus, we have developed a murine model of HPV16 + HNC modifying the published CRISPR-based Somatic Oncogene kNock-In for Cancer Modeling (CRISPR-SONIC) system for precise integration of HPV oncogenes. We showed that CRISPR-SONIC knock-in of *Kras*^*G12D*^, HPV16 E6 and E7, and a luciferase reporter at the murine β-actin 3’-UTR locus could induce spontaneous buccal tumors with sarcomatous morphology under transient or selective immunosuppression. Both preventative and therapeutic pNGVL4a-CRT/E7(detox) DNA vaccination could induce HPV16 E7-specific immune response and reduce tumor growth. Furthermore, CRISPR-SONIC knock-in of HPV16 E6 and E7 with co-delivery of HNC-relevant oncogenes *AKT* and *c-Myc* produced tumors in NSG mice capturing the characteristic carcinomic morphology of HPV + HNC. Overall, our model offers a robust platform for evaluating new therapies and exploring HPV-related carcinogenesis.

**Supplementary Information:**

The online version contains supplementary material available at 10.1186/s13578-025-01427-5.

## Background

Human papillomavirus (HPV) is the most common sexually transmitted infection, affecting approximately 80% of individuals during their lifetime [1, 2]. These DNA viruses typically result in transient infections in the skin and mucosal membranes, where the virus maintains its genome episomally [3]. However, persistent infection with high-risk HPV (HR-HPV) types, including HPV types 16 and 18, can lead to carcinogenesis [4], and integration of viral DNA into the host genome is thought to play a critical role. Specifically, viral oncogenes E6 and E7 are maintained in HR-HPV integration [5], and contribute to cellular dysregulation by inactivating the tumor suppressors p53 and pRb respectively [2]. E6 and E7 are highly expressed post-integration, which is thought to result from two main factors: disruption of the viral protein E2, which normally inhibits their expression [6], and increased stability of the resulting spliced viral-cellular fusion transcripts [7, 8]. Sequencing studies have revealed high frequency of integration in HPV-related cancers, including reports of up to 71% in HPV-associated head and neck cancer (HPV + HNC) [5, 9] and 62–98% in cervical carcinomas [10–13]. Furthermore, the presence of integration in HPV + cancers has been correlated with decreased overall survival and low immune infiltration in patients, indicating its clinical significance [5, 14].

In recent years, HPV + HNC has become a rising global epidemic [15], surpassing cervical cancer to become the most common HPV-related malignancy in the United States and other high-income countries [1]. Existing prophylactic vaccinations for HR-HPV have shown efficacy in reducing oral HPV infections (by an estimated 88.2% among vaccinated young adults in the U.S.) [16] and HPV vaccination coverage among adolescents has been on the rise (75.1% with ≥ 1 dose and 58.6% up to date among 13–17 year olds in the U.S. in 2020) [17]. Despite this, prophylactic HPV vaccinations cannot clear existing infections and progression of persistent oral HR-HPV infections to HPV + HNC remains a concern among older adults [1, 18], with incidence of oropharyngeal cancer among adults aged 70–83 projected to increase from 16.8 to 29.0 per 100,000 population by 2045 [19]. Furthermore, current treatment options for oral cancers typically include surgical resection or chemoradiotherapy [20, 21], which can reduce quality of life and potentially cause irreparable anatomical damage [22]. Therefore, it remains of critical concern to develop interventions for HPV + HNC, as well as to improve diagnostic and preventative strategies.

Although a number of preclinical models have been developed for understanding HPV-related cancer progression and therapeutic testing, it has proven difficult to fully capture disease progression as papillomaviruses are species-specific and have a life cycle reliant on stratified squamous epithelial differentiation [23]. Traditionally, humanized preclinical models are formed through xenograft implantations of HPV + tumor cell lines directly into mice. This method can generate tumors rapidly, but has some limitations including the adaptation and homogenization of cell lines under culture as well as the requirement for severe immunodeficiency [24]. The direct implantation of cell lines also renders these models incapable of capturing the distinct process of cellular transformation in HPV-related disease progression, which is vital in establishing the immune microenvironment. Thus, these orthotopic models often fail to model the human tumor microenvironment and clinical response. Another frequently used preclinical model is the transgenic mouse, created by insertion of HPV oncogenes at the embryonic level [25]. Although inclusion of a tissue-specific promoter such as K14 could limit expression of the inserted oncogenes to keratinocytes, expression would occur tissue-wide rather than focally as in an authentic HPV infection, complicating therapeutic testing [26, 27]. Furthermore, while transgenic mice are capable of immunocompetency, prenatal HPV oncoprotein expression can cause immunotolerance to introduced exogenous genes [28, 29]. Transgenic mice therefore cannot fully model the natural immune environment, rendering them less appropriate for testing immunotherapies. Conditional and inducible genetic models employing Cre recombinase have also been developed to study HPV-associated neoplasia, offering spatial and temporal control of oncogene expression [30–33]. Despite this, both conditional and conventional transgenic models are time-consuming and costly to generate, requiring initial genetic modification during early development and subsequent breeding. This makes these models impractical for efficiently exploring the variability of HPV integration across the genome. To address these challenges, we had previously developed a spontaneous murine model of HPV + buccal tumor, which could be controlled with an HPV DNA vaccine that generated a potent HPV16 E7-specific immune response [34]. This model utilized the Sleeping Beauty (SB) transposase-based system which enables rapid tumor formation through semi-random integration of oncogenes into the host genome.

HPV integration into the genome is believed to occur in a near-random manner [35], and our previously developed SB system reflects this integration process. However, recent multi-omics studies have shown evidence that there exists strong selective pressure for productive integrations, which produce actively transcribed viral-host fusion transcripts associated with higher expression of E6/E7 oncogenes compared to episomally maintained viral DNA [36, 37]. These productive integrations have been reported to occur within gene regions (introns and exons), common fragile sites, enhancers, and specific repetitive elements including Alu regions [5, 9, 14, 36, 38]. In comparison to silent (non-transcribed) integrations which are indeed generally randomly distributed, productive integrations are non-randomly distributed across viral and host genomes and result in increased tumor aggressiveness and immune evasion [36, 37]. This suggests the existence of selective pressure for productive sites, allowing for clonal expansion and tumor progression [36, 37]. Furthermore, integration typically occurs at just one or a few loci within a lesion, almost always with amplification of the host genome [39, 40]. For instance, RNA sequencing data revealed integration in cervical and HNC tumors generally resulted in only a single transcriptionally active driver integration locus [41].

Thus, we sought to generate a model of HPV + head and neck cancer with targeted oncogene insertion. To do so, we utilized the published CRISPR-based Somatic Oncogene kNock-In for Cancer Modeling (CRISPR-SONIC) system [42]. This system employs the CRISPR/Cas9 gene editing system, which uses the Cas9 enzyme to generate double-stranded breaks (DSBs) at a targeted site in the genome through use of a simple single guide RNA (sgRNA). While gene integration is typically achieved utilizing homology-directed DNA repair (HDR) with the inclusion of a homologous template, this method is limited in utility and scope due to its low efficiency. Therefore, the CRISPR-SONIC system leverages the more efficient homology-independent repair strategy, non-homologous end joining (NHEJ) [42, 43]. While this method is more error-prone, CRISPR-SONIC mitigates the impact of small indels often generated by NHEJ during DSB repair by including an internal ribosomal entry site (IRES), ensuring proper gene expression occurs [42]. Previously, Mou et al. used CRISPR-SONIC to model liver cancer, and reported a significant increase in integration efficiency compared to HDR knock-in, from 0.5% to 10% [42].

Here, we have utilized the CRISPR-SONIC system to generate a model of HPV16 + HNC which recapitulates targeted HPV integration at a single locus. HPV16 is well-suited for this model as it is the most prevalent among the HR-HPV types found in HPV + cancers, in over 50% of cervical cancers and 90–95% of HPV + HNCs [4], as well as presents the lowest clearance rate [44]. To induce malignant transformation, which typically necessitates three or more driver gene mutations or “hits” [45, 46], we modified the CRISPR-SONIC system to include HPV16 oncogenes E6 and E7 alongside the oncogenic Ras isoform *Kras*^*G12D*^. These genes were delivered with a luciferase reporter sequence to the 3’-UTR of β-actin, leveraging the robust endogenous mouse β-actin promoter for expression. In vitro transfection of murine cells confirmed the successful integration and expression of oncogenes via our Kras + HPV + SONIC system. We also showed the system was functional in vivo, generating spontaneous HPV + buccal tumors when delivered to the buccal mucosa of mice via injection and electroporation. Moreover, these tumors could be controlled by an HPV16 E7-specific immune response induced by a therapeutic HPV DNA vaccine pNGVL4a-CRT/E7 (detox). Finally, spontaneous HPV + buccal tumors modeling the reported hyperactive PI3K/AKT/mTOR pathway in HNC [39, 47–49] could be generated through CRISPR-SONIC based delivery of HPV16 E6/E7 together with oncogenes *c-Myc* and *AKT* delivered via SB100X transposase. Tumor histology revealed characteristics consistent with carcinoma, validating its clinical relevance [50–52].

## Methods

### Plasmid vectors

The Kras + HPV + SONIC donor vector SONIC-Kras^G12D^-Luc-E7-E6 was generated as follows: the Luc-T2a-E7-T2a-E6 sequence was obtained by PCR amplification of the Pkt2-Luc-T2a-E7-T2a-E6 plasmid (constructed as previously described [34]) with the following primers: 5′-AAAGCCACCATGGAAGACGCCAAAAACAT -3′ and 5′- AAAGTCGACTTACAGCTGGGTTTCTCTACGT -3′. The sequence was then cloned into the NcolI/SalI sites of the SONIC-Kras^G12D^-IRES-luciferase donor plasmid purchased from Addgene, Watertown, MA, USA (Addgene #138178) (constructed as previously described [42]). Plasmid constructs were confirmed by DNA sequencing.

The HPV + SONIC donor vector SONIC-Luc-E7-E6 was generated as follows: the Luc-T2a-E7-T2a-E6 sequence was obtained by PCR amplification of the Pkt2-Luc-T2a-E7-T2a-E6 plasmid with the following primers: 5′-AAATGGCCAATGGAAGACGCCAAAAACAT -3′ and 5′- AAAATCGATTTACAGCTGGGTTTCTCTACGT -3′. The amplified product was then cloned into the MscI/ClaI sites of the H517 SONIC HRASV12 donor plasmid purchased from Addgene (Addgene #138177) (constructed as previously described [42]). Plasmid constructs were confirmed by DNA sequencing.

The PKT2-cMyc plasmid was constructed by cloning mouse *c-Myc* into the XbaI/Bgl II sites of the PKT2/CLP-AKT vector as previously described [53]. The pcDNA3-E7/GFP plasmid was constructed by cloning HPV16 E7 into the EcoRI/BamHI sites of pcDNA3-GFP as previously described [54].

The plasmids H513 pX330.sgActin.UTR.2 (Addgene #138176), and sgA (Addgene #83807) were purchased from Addgene, developed as previously described [42]. The plasmids pT/Caggs-NRasV12 (Addgene #20205) and PKT2/CLP-AKT (Addgene #20281) were purchased from Addgene, developed as previously described [55, 56]. The plasmid pCMV(CAT)T7-SB100 (Addgene #34,879) was purchased from Addgene, developed as previously described [57].

### Cell lines

TC-1 cells (primary murine lung cells immortalized with HPV16 E6 and E7 as well as human *HRAS* oncogenes as described previously [58, 59]) were cultured in RPMI complete media supplemented with 10% fetal bovine serum, 1 mM sodium pyruvate, 2 mM l-glutamine, 100 U/ml penicillin, 100 µg/ml streptomycin, 2 mM non-essential amino acids, and 50 µM 2β-mercaptoethanol and incubated at 37 °C with 5% CO_2_.

### Animal care

Female C57BL/6 mice (6–8 weeks old) and female NSG mice (6–8 weeks old) were purchased from Jackson Laboratory and housed at the Johns Hopkins University School of Medicine Animal Facility (Baltimore, MD, USA) under pathogen-free conditions. The mice were managed in accordance to protocols by the Johns Hopkins Institutional Animal Care and Use Committee, and all efforts were made to minimize animal suffering.

### Transfection in vitro

TC-1 cells were seeded in 24-well plates and grown to 30% confluency. ExpiFectamine 293 Reagent (Gibco, Waltham, MA) was used according to manufacturer instructions to transfect the cells with a total of 5 μg of DNA per transfection, distributed as follows: 1:1:1 ratio of SONIC-Kras^G12D^-Luc-E7-E6 donor, H513 pX330.sgActin.UTR.2, and sgA; or 1:1 ratio of SONIC-Kras^G12D^-Luc-E7-E6 donor and pcDNA3-E7/GFP; or 1:1 ratio of Pkt2-Luc-T2a-E7-T2a-E6 and pCMV(CAT)T7-SB100; or 1:1 ratio of Pkt2-Luc-T2a-E7-T2a-E6 and pcDNA3-E7/GFP. Wells were imaged 3 days post-transfection using the IVIS Spectrum imager series 2000 (Xenogen, Alameda, CA, USA) after addition of D-luciferin (GoldBio, St. Louis, MO, USA). Cells from transfected wells were re-seeded and imaged again after growth for an additional 4 days. IVIS Spectrum bioluminescent images were analyzed with Living Image software (Xenogen, Alameda, CA, USA).

### Buccal tumor formation in vivo

#### Transient immune suppression

C57BL/6 mice *(n* = *5)* were transiently immunosuppressed with intraperitoneal (i.p.) injections of either 100 μg of monoclonal anti-CD3 (clone 17A2, Bio X Cell, Lebanon, NH, USA) or 100 μg of monoclonal anti-CD4 (clone GK1.5, Bio X Cell) according to the schedules shown in Fig. S1B, 2A, 3A, 4A, and 5A.

#### Buccal tumor generation

For generation of *Kras*^*G12D*^ and HPV16-E6/E7 expressing buccal tumor, C56BL/6 mice *(n* = *5)* were first anesthetized with intramuscular (i.m.) injection of ketamine/xylazine (5:1 ratio). The mice were then submucosally injected in the right buccal mucosa with the various plasmid formulations for oncogene integration, followed by invasive electroporation using the ECM 830 Square Wave Electroporation System (BTX, Holliston, MA, USA) at the injection site (8 pulses at 72v for 20 ms/pulse, with 200 ms interval between each). Fig. S1A indicates the plasmid formulations (10 μg/plasmid/mouse) for the groups (n = 5) of mice as follows: **Kras + HPV + SONIC system** *[SONIC-Kras*^*G12D*^*-Luc-E7-E6 donor, H513 pX330.sgActin.UTR.2, and sgA]* or **Nras + HPV + SB system** *[Pkt2-Luc-T2a-E7-T2a-E6, pT/Caggs-NRasV12, and pCMV(CAT)T7-SB100].*

For generation of *c-Myc*, *AKT*, and HPV-E6/E7 expressing buccal tumor, NSG mice *(n* = *3)* were anesthetized and submucosally injected at the buccal site with the **HPV + SONIC system co-delivered with AKT and c-Myc oncogenes** containing the following plasmid formulation (10 μg/plasmid/mouse): *SONIC -Luc-E7-E6 donor, H513 pX330.sgActin.UTR.2, sgA, PKT2-cMyc, PKT2/CLP-AKT, pCMV(CAT)T7-SB100* (Fig. 6A). The mice then received electroporation at the injection site (8 pulses at 72v for 20 ms/pulse, with 200 ms interval between each).

#### Vaccination

To evaluate tumor response to immunotherapy, mice were vaccinated with the DNA vaccine pNGVL4a-CRT/E7 (detox) generated as previously described [59, 60]. The vaccine (10 μg/mouse) was injected i.m. into the hind leg, followed by electroporation at the injection site (8 pulses at 106 V for 20 ms/pulse, with 200 ms interval between each) according to the schedules shown in Fig. 3A, 4A, and 5A. The E7-specific immune response induced by vaccination was evaluated by assessing E7-specific CD8 T cells in the peripheral blood via flow cytometry.

#### Flow cytometry

To evaluate immune response in vaccination experiments, quantification of E7-specific CD8 T cells was performed via flow cytometry of peripheral blood mononuclear cells (PBMCs). 10 drops of blood were drawn from the facial vein of each mouse into a tube coated with 100 μl of 10% EDTA. Blood lysis buffer (Cell Signaling Technology, Danvers, MA, USA) was added to samples to lyse red blood cells, followed by washing with 1X FACS buffer (0.5% BSA/PBS). Samples were stained with Zombie Aqua Live/Dead (#423102 BioLegend, San Diego, CA, USA). Prior to antibody staining, cells were washed and Fc blocking was done (Fc block, BD Pharmingen, San Diego, CA). Cells were then simultaneously stained at 4 °C with fluorescently conjugated APC/Fire™ 750 anti-mouse CD8a antibody (clone 53–6.7, #100766 Biolegend) and PE-conjugated HPV16 E7aa49-57 peptide loaded H-2Db tetramer (provided by the NIAID tetramer core facility, Atlanta, GA, USA). The CD8a antibody clone 53–6.7 was utilized for the tetramer staining protocol due to its ability to enhance rather than block tetramer staining [61, 62]. Following washing and resuspension in FACS buffer, flow cytometry was performed in the 13-color CytoFLEX S (Beckman Coulter, Brea, CA, USA). The data was then analyzed using FlowJo 10.4 software (BD Biosciences, Franklin Lakes, NJ, USA).

#### Bioluminescence imaging

Tumor growth in mice was quantified by bioluminescence imaging. Prior to conducting imaging, mice were anesthetized with i.m. injection of ketamine (40 mg/kg) and xylazine (0.5 mg/kg) solution (Phoenix Pharmaceutical Inc., St. Joseph, MO, USA) followed by i.p. injection of 200 µL of 3.9 mg/mL D-luciferin (GoldBio, St. Louis, MO, USA). 10 min post-injection with luciferin, mice were placed in the IVIS Spectrum imager and bioluminescence imaging was conducted. Bioluminescence signals were subsequently analyzed with Living Image software through quantification of photon flux from regions of interest.

In order to minimize mouse suffering, mouse death by tumor burden was defined as previously described, according to the following conditions: (1) bioluminescent signal (average radiance) of 10^9^ p/sec/cm^2^/sr; (2) tumor volume of 150 mm^3^; (3) 10% reduction in weight compared to average weight of healthy mice of the same age. Mice were euthanized by CO_2_ asphyxiation upon observation of these conditions.

#### Tumor marker validation

Buccal tumors excised following mouse sacrifice were initially frozen at − 80 °C. These were then placed in Trizol solution (#15596018 Invitrogen, Carlsbad, CA, USA) in a gentleMACS M Tube (#130-093-236 Miltenyi Biotec, Auburn, CA, USA), and dissociated using program RNA_02 in the gentleMACS Dissociator (#130-093-235 Miltenyi Biotec). RNA isolation was performed as follows: chloroform was added to induce phase separation, followed by collection of the RNA phase. RNA was precipitated in isopropanol, followed by washing in 75% ethanol. RNA pellets were air-dried and resuspended in RNase-free ddH_2_O. RNA yields were measured by nanodrop. RNA samples were then treated with DNase and reverse transcribed into cDNA according to manufacturer instructions (QuantiTect Reverse Transcription Kit, #205311 Qiagen, Hilden, Germany). PCR was performed to verify presence of HPV16-E6 and HPV16-E7 proteins using the following primers: HPV16-E6 forward 5’-CCACAGGAGCGACCCAGAAAG-3’ and reverse 5’-TCTGCAACAAGACATACATCG-3’, HPV16-E7 forward 5’-TATGTTAGATTTGCAACCAGA-3’ and reverse 5’-TTCCTAGTGTGCCCATTAACA-3’, and internal control β -actin forward 5′-AGGTGTGCACCTTTTATTGGTCTCAA-3′ and reverse 5′-TGTATGAAGGTTTGGTCTCCCT-3′. Amplified fragments were visualized using 6X gel loading dye (#B7024S New England BioLabs, Ipswich, MA, USA) on a 1% agarose gel with ethidium bromide (Supplementary Fig. S5).

#### Tumor histology

Buccal tumors were excised following mouse sacrifice, and fixed in 10% neutral buffered formalin for 48 h. Formalin-fixed samples were paraffin embedded and sections were stained with hematoxylin and eosin (H&E) by the Johns Hopkins University Oncology Tissue Services. The histology slides were reviewed by Dr. T.-C. Wu, a board-certified gynecologic pathologist at the Johns Hopkins University School of Medicine, Department of Pathology.

#### Tumor immunohistochemical staining

Immunostaining for c-Myc, AKT, Ki67, CK19. KRAS-G12D was performed at the Oncology Tissue Services Core of Johns Hopkins University. Immunolabeling was performed on formalin‐fixed, paraffin embedded sections on a Ventana Discovery Ultra autostainer (Roche Diagnostics). Briefly, following dewaxing and rehydration on board, epitope retrieval was performed using Ventana Ultra CC1 buffer (catalog# 6414575001, Roche Diagnostics) at with temperature and incubation time optimized for each antibody.

Primary antibodies (anti‐cMyc, 1:200 dilution, catalog# ab32072, Abcam; anti-AKT, 1:200 dilution, catalog # 10176-2-AP, Proteintech; anti‐Ki67, 1:200 dilution, catalog# Ab16667, Abcam; anti‐CK19, 1:800 dilution; catalog# ab133496, Abcam; anti-KRAS-G12D, 1:75 dilution, catalog# MA5-36256, ThermoFischer) were applied at 36–37 °C for 60 min. Primary antibodies were detected using an anti-rabbit HQ detection system (catalog# 7017936001 and 7017812001, Roche Diagnostics) followed by Chromomap DAB IHC detection kit (catalog # 5266645001, Roche Diagnostics), counterstaining with Mayer’s hematoxylin, dehydration and mounting.

Immunostaining for OSCAR (catalog# 300 M-18 predilute antibody, SigmaAldrich) was performed in the clinical histology lab on the Benchmark XT autostainer (Ventana Medical Systems Inc., Tucson, AZ) using the I-View detection kit and standard methods.

### Statistical analysis

GraphPad PRISM 9 Software (GraphPad, Boston, MA, USA) was used for statistical analysis, and data was expressed as the mean ± the standard error of the mean (SEM). One-way ANOVA with Tukey–Kramer multiple comparisons test or Student’s t-test was used to determine statistical significance. To measure survival outcomes, Kaplan–Meier plots of survival percentage were constructed and the log-rank test was used to determine whether survival times between groups displayed significant differences. In all cases, p-values ≤ 0.05 were considered statistically significant.

## Results

### CRISPR-SONIC system is required for targeted integration and efficient expression from SONIC donor construct in vitro in TC-1 cells

To enable modeling of HPV integration and tumorigenesis, we modified the single-cut donor vector of the CRISPR-SONIC system to deliver HPV16 E6/E7, *Kras*^*G12D*^, and luciferase. The recombinant SONIC-Kras^G12D^-Luc-E7-E6 donor vector was paired with (1) a plasmid **H513 pX330.sgActin.UTR.2** encoding the Cas9 enzyme and sgRNA targeting the host’s β-actin site, and (2) a plasmid **sgA** encoding sgRNA targeting the sgA site on the donor plasmid for the purpose of linearization [42]. Upon delivery of this system, knock-in occurs through NHEJ, enabling insertion of the linearized donor into the cleaved 3’-UTR of β-actin. Integration specificity and efficacy at the β -actin 3’ UTR using this single-cut system with sgRNA targeting the β -actin 3’ UTR were previously confirmed by Mou et al. via Sanger sequencing of junction amplicons [42]. Here, we leveraged this validated platform with a modified payload to model HPV integration and tumorigenesis. When properly oriented, the Kras^G12D^-Luc-E7-E6 sequence would be expressed under the control of the β-actin promoter and IRES signal and be subsequently terminated by the poly A signal. We also compared the activity of this Kras + HPV + SONIC system to our previously developed Nras + HPV + SB system [34], which utilizes the SB100X hyperactive transposase to deliver oncogenes HPV16 E6/E7 and *Nras*.

**Fig. 1:**
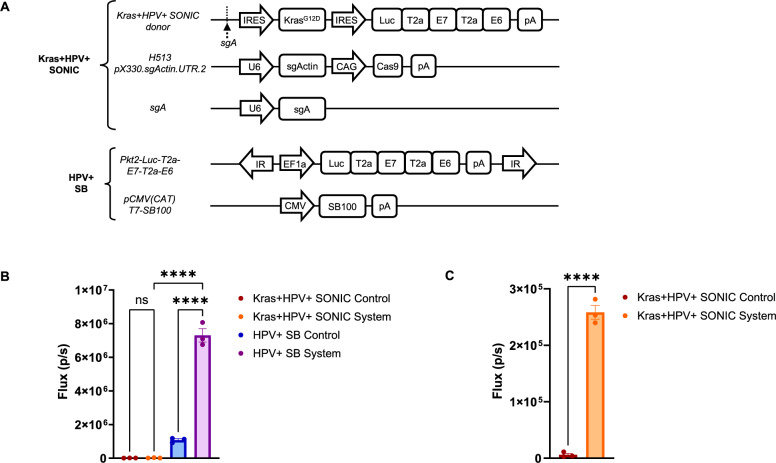
CRISPR-SONIC system is required for targeted integration and efficient expression from SONIC donor construct in vitro in TC-1 cells**.** In vitro integration of *Kras*^*G12D*^, HPV oncogenes, and luciferase reporter into murine cells via Kras + HPV + SONIC or HPV + SB system. **A** Plasmids used for transfection of TC-1 cells. Integration-deficient controls for the Kras + HPV + SONIC system and HPV + SB system were generated by replacing the H513 pX330.sgActin.UTR.2 and sgA plasmids, as well as the pCMV(CAT)T7-SB100 plasmid respectively with pcDNA3-E7/GFP for dosage balance. Bioluminescence signals were quantified by imaging on the IVIS Spectrum imager after addition of luciferin. **B** Bioluminescence signals of TC-1 cells transfected with Kras + HPV + SONIC system, Kras + HPV + SONIC control, HPV + SB system, or HPV + SB control at 3 days post-transfection. Data represent the mean ± SEM of 3 replicates. One-way ANOVA analysis was conducted followed by the Tukey–Kramer Test. **C** Bioluminescence signals of TC-1 cells transfected with Kras + HPV + SONIC system or Kras + HPV + SONIC control at 7 days post-transfection. Data represent the mean ± SEM of 3 replicates. Unpaired two-tailed t-test analysis was conducted. ****p < 0.0001 vs control with a p ≤ 0.05 considered statistically significant

To validate the functionality of the Kras + HPV + SONIC system and compare its activity to the SB system, TC-1 cells were transfected with either **(1) Kras + HPV + SONIC system [SONIC-Kras**^*G12D*^*-Luc-E7-E6 donor, H513 pX330.sgActin.UTR.2, and sgA]*; **(2) Kras + HPV + SONIC control** *[SONIC-Kras*^*G12D*^*-Luc-E7-E6 donor and pcDNA3-E7/GFP];* **(3) HPV + SB system** *[Pkt2-Luc-T2a-E7-T2a-E6 and pCMV(CAT)T7-SB100];* **(4) HPV + SB control** *[Pkt2-Luc-T2a-E7-T2a-E6 and pcDNA3-E7/GFP]*; where **(2)** and **(4)** are negative controls replacing required constituents for gene insertion by the SONIC and SB system respectively with a pcDNA3-E7/GFP vector control for dosage balance (Fig. 1A). Bioluminescence signals were measured in the IVIS Spectrum imager following addition of luciferin, at 3- and 7-days post-transfection. The Kras + HPV + CRISPR-SONIC system displayed no difference in signal compared to its paired control at 3 days post-transfection (Fig. 1B), but demonstrated significantly higher signal *(p* < *0.0001)* compared to its control at 7 days post-transfection (Fig. 1C). This result suggests successful integration and expression from the donor plasmid by the Kras + HPV + SONIC system.

In contrast, a significantly higher *(p* < *0.0001)* luminescence signal in the HPV + SB system was seen by 3 days post-transfection compared to the negative controls and the Kras + HPV + SONIC system. (Fig. 1B). Low levels of basal expression in the HPV + SB negative control were also seen despite the lack of the transposase plasmid. This suggests the SB system leads to higher expression levels from the *Pkt2-Luc-T2a-E7-T2a-E6* plasmid compared to the expression from the *SONIC-Kras*^*G12D*^*-Luc-E7-E6* donor plasmid with the SONIC system.

### Kras + HPV + SONIC system generates spontaneous HPV + buccal tumors in C57BL/6 mice with transient immune depletion

#### Transient CD3 T cell depletion enables tumor formation

We previously demonstrated that spontaneous formation of tumors required immunosuppression, with optimal tumor formation via CD3 depletion [34]. It was shown that transient depletion with anti-CD3 prior to oncogenic plasmid transfection could enable tumor induction and growth while also allowing recovery of the CD3 T cell population over time [34].

**Fig. 2:**
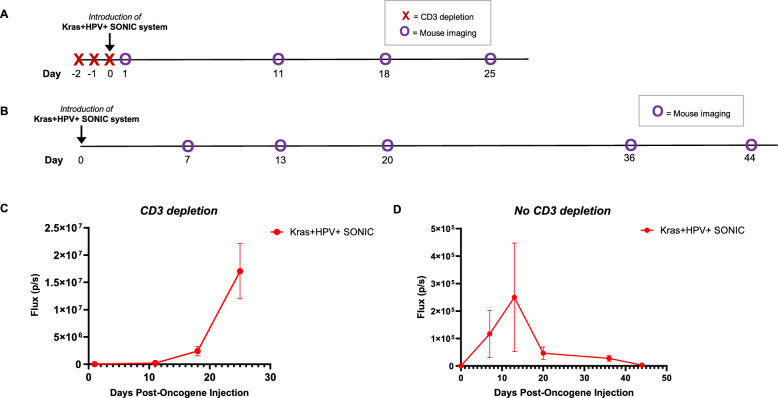
Kras + HPV + SONIC system generates spontaneous HPV + buccal cancer in C57BL/6 mice with transient immune depletion. **A** Schedule of CD3 depletion and bioluminescence imaging of buccal tumors in a group of mice (n = 5). Kras + HPV + SONIC was introduced in the buccal region of mice through submucosal injection and electroporation on Day 0, as indicated. Tumor growth by bioluminescence signal was quantified by imaging on the IVIS Spectrum after intraperitoneal injection of luciferin. **B** Schedule of Kras + HPV + SONIC system introduction in immunocompetent mice (n = 5) and subsequent bioluminescence imaging. **C** Quantified bioluminescence kinetics of buccal tumors in mice with an initial 3-day anti-CD3 administration prior to administration of Kras + HPV + SONIC system. **D** Quantified bioluminescence kinetics of buccal tumors in immunocompetent mice administered the Kras + HPV + SONIC system

Thus, to generate spontaneous buccal tumors in mice, two groups of C57BL/6 mice *(n* = *5)* were injected intraperitoneally (i.p.) with anti-CD3 daily for 3 days, and plasmids were introduced through submucosal injection at the buccal site followed by electroporation on the third day. As depicted in Fig. S1A, the administered plasmids included: **(1) Kras + HPV + SONIC system** *[SONIC-Kras*^*G12D*^*-Luc-E7-E6 donor, H513 pX330.sgActin.UTR.2, and sgA]*; and **(2) Nras + HPV + SB system** *[Pkt2-Luc-T2a-E7-T2a-E6, pT/Caggs-NRasV12, and pCMV(CAT)T7-SB100].* As tumor growth was previously shown to correlate with bioluminescence signal [34, 42], mice were imaged weekly in the IVIS Spectrum following i.p. injection of luciferin to measure signal from buccal tumors (Fig. 2A).

The Kras + HPV + SONIC system was capable of inducing spontaneous buccal tumor growth in the transiently immunosuppressed mice, as indicated by increase in luminescence intensity over time (Fig. 2C). In comparison, mice with tumors induced by the Nras + HPV + SB system showed significantly higher luminescence by Week 1 *(p* < *0.001)* than with the Kras + HPV + SONIC system (Supplementary Fig. S1C). However, by the second week there was no significant difference in luminescence signal between the two groups. Furthermore, mice injected with the Nras + HPV + SB system plasmids experienced lower survival percentages compared to the Kras + HPV + SONIC system (Supplementary Fig. S2C). These results suggest that while the Nras + HPV + SB system leads to higher expression levels in tumors, the Kras + HPV + SONIC system effectively induces spontaneous tumor formation with controlled expression and lower mortality.

#### Spontaneous tumor growth is not supported in immunocompetent mice

To confirm whether transient immunosuppression was required for spontaneous tumor formation, administration of the Kras + HPV + SONIC system was also performed without prior CD3 T cell depletion in fully immunocompetent C57BL/6 mice (Fig. 2B). Tumor growth, monitored by weekly luminescence signal imaging, increased for two weeks followed by a rapid decline (Fig. 2D). These results further support the need for temporary immune suppression, as demonstrated through transient CD3-depletion here, in order to generate spontaneous HPV + buccal tumors.

### Kras + HPV + SONIC-induced tumors can be controlled by therapeutic DNA vaccination with pNGVL4a-CRT/E7 (detox)

To assess whether Kras + HPV + SONIC-induced buccal tumors were capable of responding to immunotherapy, mice were vaccinated with the DNA vaccine pNGVL4a-CRT/E7 (detox), which expresses human calreticulin (CRT) and detoxified HPV16-E7 antigen as previously described [59, 60].

**Fig. 3:**
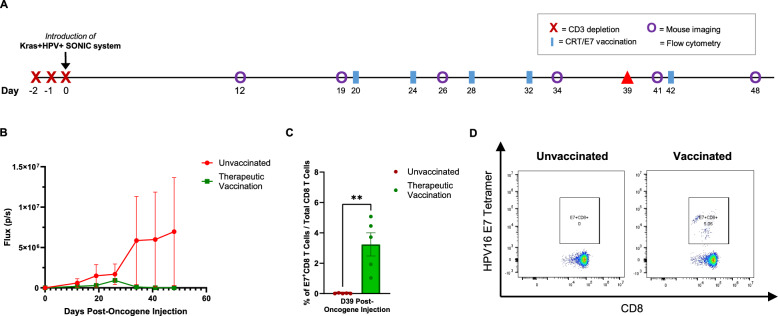
Therapeutic vaccination induces HPV16 E7-specific CD8 T cell response and regression of Kras + HPV + SONIC-induced spontaneous buccal tumors in CD3-depleted C57BL/6 mice. **A** Schedule of CD3 depletion, introduction of Kras + HPV + SONIC system, bioluminescence imaging of buccal tumors, and flow cytometry analysis of PBMCs for quantification of tumor-specific immune response in 2 groups of mice (n = 5). Therapeutic vaccination with pNGVL4a-CRT/E7(detox) DNA vaccine was performed as indicated in one group of CD3-depleted mice. The remaining group of CD3-depleted mice did not receive vaccination. **B** Quantified bioluminescence kinetics of buccal tumors in CD3-depleted mice, either with or without therapeutic vaccination. **C** Flow cytometry of PBMCs collected at Day 39 post-oncogene injection from either vaccinated or unvaccianted mice was performed to determine the proportion of E7-specific CD8 T cells out of total CD8 T cells. **D** Representative flow cytometry images of E7-specific CD8 T cells gated out of total CD8 T cells in either vaccinated or unvaccinated mice administered anti-CD3. Data represent the means ± SEM. Unpaired two-tailed t-test was conducted with a p ≤ 0.05 considered statistically significant (**p < 0.01 vs control)

Two groups of C57BL/6 mice *(n* = *5)* were given 3 days of anti-CD3 administration prior to introduction of the Kras + HPV + SONIC system to the buccal region (Fig. 3A). After buccal tumors formed as indicated by bioluminescence imaging, one group of mice was vaccinated in 4-day intervals for a total of four vaccinations, followed by an additional fifth booster vaccination at Day 42 post-oncogene injection (Fig. 3A). While the unvaccinated group showed a continuous upward trend in bioluminescence signal, the vaccinated group saw bioluminescence signal return to baseline levels, indicating tumor regression (Fig. 3B). In addition to weekly bioluminescence imaging to track tumor growth, blood was also collected from the mouse facial vein to analyze the immune response to vaccination. Flow cytometry indicated that the E7-specific CD8 T cell population increased significantly in response to vaccination compared to in the unvaccinated group (Fig. 3C, D). These results indicate Kras + HPV + SONIC-induced tumors can be effectively controlled by vaccination, with tumor regression correlated to a robust HPV16 E7-specific CD8 T cell response, demonstrating the potential of this model for evaluating immunotherapeutic strategies.

### pNGVL4a-CRT/E7 (detox) DNA vaccination controls Kras + HPV + SONIC-induced buccal tumors in a CD4 T cell-compromised model

Additionally, to mimic the CD4 T cell-compromised conditions seen in immunocompromised hosts such as HIV patients, for whom HPV infections are of concern, tumors were also induced in mice undergoing continuous CD4 depletion. As the pNGVL4a-CRT/E7 (detox) vaccine was previously described to effectively target HPV16 E6/E7 expressing tumors in the absence of CD4 T cells in other models of HPV + cancer [63], the ability of the Kras + HPV + SONIC-induced tumors to respond to the vaccination in a CD4-deficient environment was also examined here.

#### Therapeutic vaccination generates HPV16 E7-specific immune response and tumor control in CD4-depleted mice

**Fig. 4:**
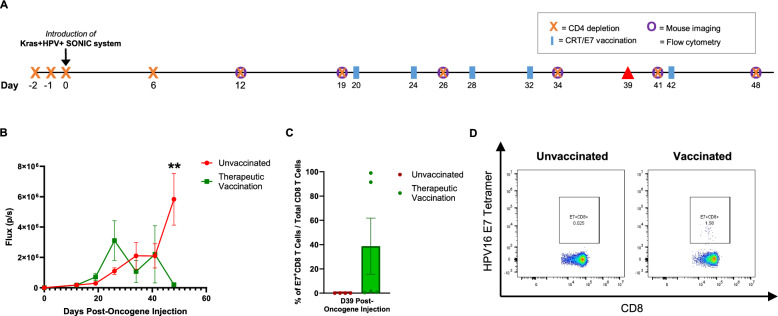
Therapeutic vaccination induces HPV16 E7-specific CD8 T cell response and regression of Kras + HPV + SONIC-induced spontaneous buccal tumors in CD4-depleted C57BL/6 mice.** A** Schedule of CD4 depletion, introduction of Kras + HPV + SONIC system, bioluminescence imaging of buccal tumors, and flow cytometry analysis of PBMCs for quantification of tumor-specific immune response in 2 groups of mice (n = 5). Therapeutic vaccination with pNGVL4a-CRT/E7(detox) DNA vaccine was performed as indicated in one group of CD4-depleted mice. The remaining group of CD4-depleted mice did not receive vaccination. **B** Quantified bioluminescence kinetics of buccal tumors in CD4-depleted mice, either with or without therapeutic vaccination. **C** Flow cytometry of PBMCs collected at Day 39 post-oncogene injection from either vaccinated or unvaccianted mice was performed to determine the proportion of E7-specific CD8 T cells out of total CD8 T cells. **D** Representative flow cytometry images of E7-specific CD8 T cells gated out of total CD8 T cells in either vaccinated or unvaccinated mice administered anti-CD4. Data represent the means ± SEM. Unpaired two-tailed t-test was conducted with a p ≤ 0.05 considered statistically significant (**p < 0.01 vs control)

Two groups of C57BL/6 mice *(n* = *5)* were depleted with 3 days of anti-CD4 administration prior to Kras + HPV + SONIC system introduction to the buccal region, followed by ongoing anti-CD4 administration weekly (Fig. 4A). After buccal tumors formed as indicated by bioluminescence imaging, one group of mice was vaccinated in 4-day intervals for a total of four vaccinations, followed by an additional fifth booster vaccination at Day 42 post-oncogene injection (Fig. 4A). Imaging revealed that tumors had significantly lower signal after vaccination compared to the unvaccinated group, where tumors continued to increase in bioluminescence intensity (Fig. 4B). In addition to weekly bioluminescence imaging to track tumor growth, blood was also collected from the mouse facial vein to analyze the immune response to vaccination. Flow cytometry indicated that vaccination of the tumor-bearing CD4-depleted mice appeared to generate a potent E7-specific CD8 T cell response, although a significant difference was not seen compared to the unvaccinated group (Fig. 4C, D). These results indicate that Kras + HPV + SONIC-induced tumors can still be controlled by vaccination correlated with a robust HPV16 E7-specific CD8 T cell response, even in the absence of CD4 T cells, highlighting the model’s relevance for immunocompromised conditions.

#### Preventative vaccination with pNGVL4a-CRT/E7 (detox) provides antitumor immunity in CD4-depleted mice

**Fig. 5:**
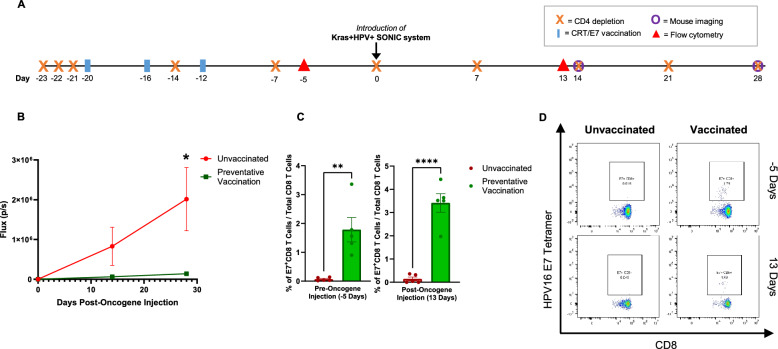
Preventative vaccination induces HPV16 E7-specific CD8 T cell response and prevents growth of Kras + HPV + SONIC-induced spontaneous buccal tumors in CD4-depleted C57BL/6 mice. **A** Schedule of CD4 depletion, introduction of Kras + HPV + SONIC system, bioluminescence imaging of buccal tumors, and flow cytometry analysis of PBMCs for quantification of tumor-specific immune response in 2 groups of mice (n = 5). Preventative vaccination with pNGVL4a-CRT/E7(detox) DNA vaccine was performed as indicated in one group of CD4-depleted mice, while the other group did not receive vaccination. **B** Quantified bioluminescence kinetics of buccal tumors in CD4-depleted mice, either with or without preventative vaccination. **C** Flow cytometry of PBMCs collected from the mice was used to determine the proportion of E7-specific CD8 T cells out of total CD8 T cells before and after introduction of oncogenes via Kras + HPV + SONIC. **D** Representative flow cytometry images of E7-specific CD8 T cells gated out of total CD8 T cells at 5 days pre-oncogene injection, and 13 days post-oncogene injection. Data represent the means ± SEM. Unpaired t-test was conducted with a p ≤ 0.05 considered statistically significant (*p < 0.05, **p < 0.01, ****p < 0.0001 vs control)

To determine if Kras + HPV + SONIC-induced buccal tumor growth could be attenuated via preventative vaccination, CD4-depleted mice (3 days of initial depletion, followed by weekly depletion thereafter) were vaccinated with pNGVL4a-CRT/E7 (detox) in 4-day intervals for a total of three times prior to introduction of oncogenes (Fig. 5A). Bioluminescence imaging revealed growth of tumors in unvaccinated mice, whereas bioluminescence signal remained significantly lower in vaccinated mice (Fig. 5B).

In addition, blood samples were collected post-vaccination prior to administration of the Kras + HPV + SONIC system, as well as after growth of the tumor. Prior to oncogene introduction, flow cytometry analysis indicated that vaccinated mice had significantly increased E7-specific CD8 T cells compared to naïve mice (Fig. 5C, D). Post oncogene integration, the amount of E7-specific CD8 T cells remained significantly higher in the vaccinated group compared to the unvaccinated group (Fig. 5C, D). These results demonstrate that preventative vaccination with pNGVL4a-CRT/E7 (detox) successfully primes the immune system in CD4-depleted mice, providing protection against Kras + HPV + SONIC-induced tumors correlated with sustained HPV16 E7-specific CD8 T cell activity.

### HPV + SONIC system in conjunction with *AKT* and *c-Myc* oncogenes generates tumors with positive epithelial markers consistent with carcinoma in NSG mice

While studies have shown significant correlation between HPV + status and *KRAS* activation and mutations in HPV + HNC clinically [64, 65], the PI3K/AKT/mTOR pathway is reported to be hyperactive and the most mutated pathway in HNC [39, 47–49]. Thus, we sought to generate a more comprehensive and clinically relevant model of HPV + HNC by utilizing the CRISPR-SONIC system to deliver HPV16 E6 and E7, along with delivery of oncogenes *c-Myc* and *AKT* via SB100X transposase.

**Fig. 6:**
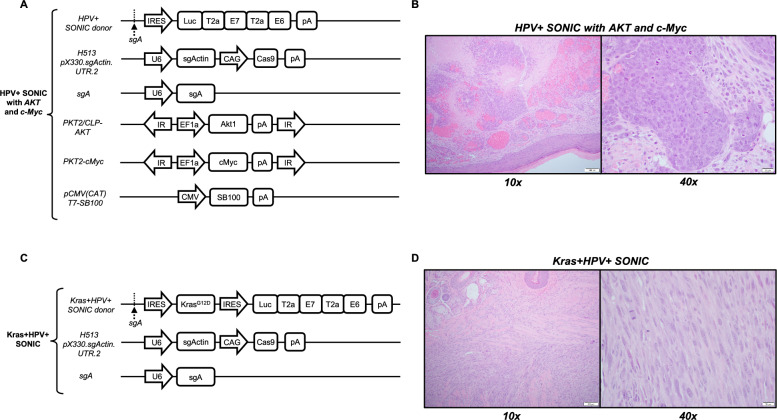
Spontaneous buccal tumors induced by HPV + SONIC system with *AKT* and *c-Myc* oncogene co-delivery in NSG mice demonstrate carcinoma morphology, while Kras + HPV + SONIC-induced buccal tumors in C57BL/6 mice demonstrate sarcoma morphology.** A** Schematic diagram of plasmids used in the HPV + SONIC system with *AKT* and *c-Myc* oncogene co-delivery for tumor generation in NSG mice (n = 3) via submucosal injection followed by electroporation. **B** Representative histological section of buccal tumor extracted from an NSG mouse administered the HPV + SONIC system with *AKT* and *c-Myc* oncogene co-delivery demonstrates carcinoma-like phenotype. Left: 10 × magnification shows dense cellularity with areas of hemorrhage and necrosis. Right: 40 × magnification reveals pleomorphic cells with prominent nucleoli and frequent mitotic figures. **C** Schematic diagram of plasmids used in Kras + HPV + SONIC system used for tumor generation in C57BL/6 mice (n = 5) via submucosal injection followed by electroporation. **D** Representative histological section of buccal tumor extracted from a C57BL/6 mouse administered Kras + HPV + SONIC system demonstrates sarcoma-like phenotype. Left: 10 × magnification displays spindle-shaped cells within the stroma. Right: 40 × magnification demonstrates elongated cells with a uniform chromatin pattern and minimal nuclear pleomorphism

**Fig. 7:**
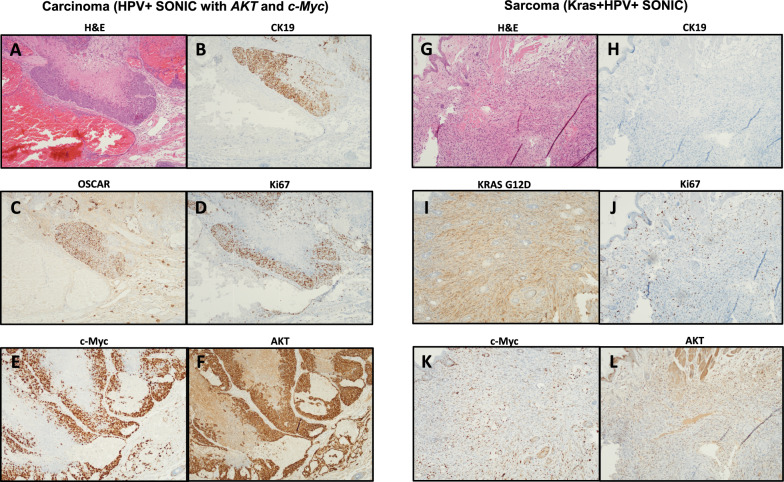
H&E histology and immunohistochemical characterization of carcinoma induced by HPV + SONIC system with AKT and c-Myc oncogene co-delivery in NSG mice (A-F) and sarcoma induced by Kras + HPV + SONIC system in C57BL/6 mice (G-L). **A** The carcinoma (generated with HPV + SONIC with *AKT* and *c-Myc*) shows a nodular growth pattern with high grade histology, central necrosis, and abundant hemorrhage on routine H&E staining. **B**, **C** Staining for CK19 and OSCAR, respectively, is present in a strong and patchy manner, supporting epithelial origin for the carcinoma. **D** Ki67, a marker of cell proliferation, is significantly elevated in the viable carcinoma. **E**, **F** Staining for c-Myc and AKT in a nuclear and membranous pattern (respectively) is strong and diffuse in the viable carcinoma, as expected given their use in creating the carcinoma model. **G** The sarcoma (generated with Kras + HPV + SONIC) demonstrates spindled and fascicular morphology with atypia and frequent mitoses on routine H&E staining. **H** CK19 is negative, as expected, for a tumor of mesenchymal origin. **I** KRAS^G12D^ is positive in the sarcomatous elements as expected, given its role in creating the tumor, while negative in the overlying skin and hair follicles. **J** Ki67 is significantly elevated in the sarcoma. **K** c-Myc is present in a similar pattern to Ki67, highlighting dividing cells and involved inflammatory cells and lymphocytes. **L** AKT demonstrates only non-specific staining in the sarcoma areas

We prepared the SONIC-Luc-E7-E6 donor plasmid and delivered it in immunodeficient NOD SCID gamma (NSG) mice *(n* = *3)* via submucosal injection to the buccal site together with plasmids H513 pX330.sgActin.UTR.2 and sgA, as well as PKT2-cMyc, PKT2/CLP-AKT, and pCMV(CAT)T7-SB100 (Fig. 6A). This formulation was able to induce tumor formation in the NSG mice (with visible tumor formation by day 90 post-oncogene delivery). Investigation with routine histologic methods revealed high-grade cytology with atypical nuclear features, high nuclear to cytoplasmic ratio, and frequent mitoses (Fig. 6B, 7A). The HPV + SONIC system in conjunction with *AKT* and *c-Myc* oncogenes displayed cohesive growth in a lobulated and nested architectural pattern with extensive necrosis and hemorrhage indicative of rapid and aggressive growth. Its epithelial origins were confirmed with immunohistochemical staining by both CK19 (Fig. 7B) and OSCAR (Fig. 7C) which are low molecular weight and broad-spectrum cytokeratin stains, respectively, in a focal to patchy pattern, consistent with the high-grade features. Ki67, an immunohistochemical marker of rapidly dividing cells, was elevated in viable tumor (Fig. 7D). The tumor also stained strongly for both c-Myc (Fig. 7E) and AKT (Fig. 7F), in a nuclear and membranous pattern respectively, as expected given the use of these genes in creating the model system.

The Kras^G12D^-expressing tumor induced by Kras + HPV + SONIC system (Fig. 6C) in C57BL/6 mice was similarly characterized and demonstrated spindled and fascicular growth with frequent mitoses on routine H&E staining (Fig. 6D, 7G). The tumor did not show expression of CK19 (Fig. 7H), supporting a non-epithelial origin. Kras^G12D^ expression, and its absence in the overlying skin and adnexal structures, was as expected given its use in inducing tumor formation (Fig. 7I). Similarly, an elevated Ki67 was expected given the frequent mitoses seen on H&E staining (Fig. 7J). While c-Myc and AKT have both been reported to be involved in pathways associated with sarcoma development, they were not used in the creation of the Kras + HPV + SONIC model, and so their staining pattern was expected to be quite different than that seen in the HPV + SONIC system in conjunction with *AKT* and *c-Myc* oncogenes. c-Myc appears to highlight tissue associated lymphocytes and inflammatory cells and only occasional sarcoma cells, as expected given its role in cell cycling and cell division (Fig. 7K). AKT staining was a diffuse blush in a cytoplasmic and nuclear, rather than membranous, pattern, indicative of a non-specific staining profile (Fig. 7L). Taken together, immunohistochemical characterization supports an epithelial origin of the tumors generated using the HPV + SONIC system in conjunction with *AKT* and *c-MYC* oncogenes consistent with carcinoma, while tumors generated using the Kras + HPV + SONIC system generated sarcoma.

## Discussion

In this study, we developed a spontaneous murine model of HPV + buccal cancer using the CRISPR-SONIC system, allowing for targeted integration of HPV oncogenes and relevant drivers into the genome. The Kras + HPV + SONIC system successfully induced tumor growth in C57BL/6 mice under transient or selective immunosuppression, and these tumors were responsive to both therapeutic and preventative immunotherapeutic vaccination, demonstrating an HPV16 E7-specific immune response. Furthermore, integration of oncogenes HPV16 E6/E7 via the SONIC system along with co-delivery of *AKT* and *c-Myc* oncogenes in NSG mice enabled formation of spontaneous carcinomic tumors with clinical and morphological relevance.

In our in vitro validation, the CRISPR-SONIC system demonstrated its ability to drive gene expression from the donor plasmid (Fig. 1C), though at lower levels than the SB system (Fig. 1B). The significantly higher signal observed with SB is in accordance with its semi-random integration mechanism and potential for multi-copy insertions. In contrast, the SONIC system facilitates targeted insertion at the β-actin 3’-UTR, limiting insertion to up to two alleles. Thus, the observed differences in signal likely reflect the inherent capabilities of each system. Interestingly, low levels of basal expression were also seen in the HPV + SB negative control despite the absence of the transposase plasmid driving integration. This may potentially be due to the presence of the strong EF1a promoter in the transposon plasmid (Fig. 1A) [66]. On the other hand, the Kras + HPV + SONIC donor plasmid lacks an intrinsic promoter and therefore requires integration into a location with an active promoter for proper expression. Given the lower viral antigen load induced via CRISPR-SONIC compared to the SB system, our CRISPR-SONIC model of HPV + HNC is relevant for providing a more stringent environment to test immunotherapies, ensuring they also function to target less immunogenic tumors such as those with fewer HPV integrations.

Following in vivo delivery, spontaneous buccal tumors formed using the Kras + HPV + SONIC system in mice required temporary or selective immunosuppression, as was previously shown to be necessary with the SB system [34]. This is potentially due to the inflammatory nature of the electroporation delivery method and the immunogenicity of the HPV16 E6, HPV16 E7, and luciferase xenoantigens. Immune response to luciferase has previously been shown to impair tumor growth and prevent spontaneous metastases in mice [67, 68]. This can potentially be addressed by utilizing mice pre-tolerized to luciferase, such as the “Glowing Head” or “NoGlow” mouse models [69, 70], to prevent pre-emptive immune clearance. Furthermore, E6 and E7 antigens are known to elicit humoral and cellular responses [71], contributing to the better survival and enhanced therapy responses observed in HPV + tumors compared to their HPV- counterparts [72, 73]. The administration of anti-CD3 and anti-CD4 antibodies for immunosuppression likely delayed immune priming, facilitating tumor formation. This parallels the virus’s documented ability to evade or modulate host immune responses during infection, contributing to HPV persistence and pathogenesis [74–78].

Our use of transient or selective immunosuppression in generating HPV + tumor models also reflects the association of immunosuppressive conditions with increased susceptibility to HPV infection and cancer in clinically relevant scenarios, particularly in cases of HIV infection, which compromises CD4 T cells [79], and organ transplantation, which necessitates immunosuppressive therapy [80, 81]. Our model effectively mimics these scenarios, especially HIV infection through continuous anti-CD4 administration to deplete CD4 T cells. Notably, despite transient CD3- or continuous CD4-depletion, a specific immune response and tumor control were still achieved through vaccination with pNGVL4a-CRT/E7(detox). Additionally, the generation of an E7-specific immune response in mice undergoing continued CD4 depletion (Fig. 4C, D, 5C, D) suggests the vaccine can function in a CD4-independent manner, supporting our earlier findings [34, 63]. This demonstrates the potential of therapeutic vaccines to elicit robust immune responses even in immunocompromised hosts, and confirms our model’s value for evaluating novel therapies.

To further the clinical relevance of our model, we generated two CRISPR-SONIC systems, both including HPV16 E6 and E7 viral oncogenes but differing in the inclusion of *Kras*^*G12D*^ versus *AKT* and *c-Myc* oncogenes. Here, inclusion of *Kras*^*G12D*^ generated a tumor morphology closer to sarcoma (Fig. 6D), while AKT and c*-*Myc bearing tumors closely resembled the more clinically-relevant carcinoma (Fig. 6B) [50–52]. As noted with our previous SB model, the sarcoma morphology may potentially be due to transformation of non-epithelial cells, and could be addressed in future studies by incorporating a K14 promoter to restrict expression to epithelial cells [34, 82, 83]. Further molecular characterization using immunohistochemistry supports an epithelial origin of the carcinoma tumors generated via the HPV + SONIC system with *AKT* and *c-Myc* oncogenes, as opposed to the development of sarcoma with the Kras + HPV + SONIC system. Future evaluation of the HPV + SONIC with *AKT* and *c-Myc* system in transiently immunosuppressed C57BL/6 mice could also render this model applicable for investigating immunotherapeutic strategies. These analyses will optimize the model for studying HPV-driven carcinomas and enhance its clinical relevance. Additionally, as HPV-driven tumorigenesis predominantly occurs in the oropharynx rather than the oral cavity [84–86], future studies could explore induction of the SONIC system through intralingual injection targeting the base of the tongue, considered part of the murine equivalent of the human oropharyngeal region [87]. Further considerations of our model include the limitations inherent with the CRISPR-SONIC method [42]. Among these include potential inverted insertion of the donor vector, possible off-target insertion through non-specific targeting or at natural DSBs, and the concern of mono- versus bi-allelic insertion at the targeted locus. As discussed by Mou et al., further optimization and investigation is warranted to address these concerns [42].

The unique advantage of our model lies in its ability to target specific genomic loci, as originally validated by Mou et al. [42], allowing the study of HPV integration hotspots. In this model, oncogene integration at the ꞵ-actin site was maintained to leverage the robust endogenous promoter for oncogene expression and induce rapid tumor generation. However, the CRISPR-SONIC system can be easily modified to target different HPV integration sites through the design of specific sgRNAs. Additionally, multiplexed gRNAs can be employed for targeting multiple genomic loci simultaneously, if desired [88]. This flexibility in targeting, combined with increasing multi-omics data on potential integration hotspots in patient tumors, gives our CRISPR-SONIC-based model of HPV + HNC exciting prospects in development of precision medicine approaches. Advances in sequencing technologies have led to greater interest in characterizing HPV integration sites and their potential role in malignant transformation, including through alteration of normal gene expression [38]. Clonal selection of aggressively expanding cells [4] has been suggested to result in strong natural selection for integration in key carcinogenic genes [5]. Integration events in cancer-related genes have also been linked to recurrent tumors, while responsive HPV + HNC tumors contained integration events mainly in intergenic loci [89]. Thus, reported integration locations which may be of interest to explore with our model include sites within or near tumor suppressor genes such as *TP63* [9, 90, 91], or near oncogenes such as *MYC* [92, 93].

## Conclusions

In conclusion, our CRISPR-SONIC based model of HPV + HNC enables precise integration of HPV oncogenes to generate spontaneous, localized tumors under transient or selective immune suppression which are capable of being controlled by immunotherapeutic vaccination. This model complements our previously developed SB system, which offers the advantage of generating a range of diverse genomic alterations and varying growth rates that could allow for therapeutic testing in a more heterogeneous population and potentially capture a wider range of treatment responses. On the other hand, our CRISPR-SONIC based model could facilitate development of more personalized treatments which have become of increasing interest due to the clinical and molecular heterogeneity of HNC. With a system to precisely integrate HPV oncogenes, it would be possible to examine potential functional roles of relevant integration hotspots in altering gene expression and contributing to carcinogenesis. This could lead to improved screening strategies and patient risk stratification tools, as well as the development and testing of more targeted treatments including immunotherapies.

## Supplementary Information

Supplementary file 1.

## Data Availability

All data relevant to the study are included in the article or uploaded as supplementary information. Data and materials are available on reasonable request.

## References

[1] Roman BR, Aragones A. Epidemiology and incidence of HPV-related cancers of the head and neck. J Surg Oncol. 2021;124:920–2. 10.1002/jso.26687. 34558067 10.1002/jso.26687 PMC8552291

[2] Anna Szymonowicz K, Chen J. Biological and clinical aspects of HPV-related cancers. Cancer Biol Med. 2020;17:864–78. 10.20892/j.issn.2095-3941.2020.0370. 33299640 10.20892/j.issn.2095-3941.2020.0370 PMC7721094

[3] Zhou L, Qiu Q, Zhou Q, Li J, Yu M, Li K, et al. Long-read sequencing unveils high-resolution HPV integration and its oncogenic progression in cervical cancer. Nat Commun. 2022;13:2563. 10.1038/s41467-022-30190-1. 35538075 10.1038/s41467-022-30190-1 PMC9091225

[4] Lace MJ, Anson JR, Klussmann JP, Wang DH, Smith EM, Haugen TH, et al. Human papillomavirus type 16 (HPV-16) genomes integrated in head and neck cancers and in HPV-16-immortalized human keratinocyte clones express chimeric virus-cell mRNAs similar to those found in cervical cancers. J Virol. 2011;85:1645–54. 10.1128/JVI.02093-10. 21123375 10.1128/JVI.02093-10 PMC3028875

[5] Koneva LA, Zhang Y, Virani S, Hall PB, McHugh JB, Chepeha DB, et al. HPV integration in HNSCC correlates with survival outcomes, immune response signatures, and candidate drivers. Mol Cancer Res. 2018;16:90–102. 10.1158/1541-7786.MCR-17-0153. 28928286 10.1158/1541-7786.MCR-17-0153 PMC5752568

[6] Sathish N, Wang X, Yuan Y. Human papillomavirus (HPV)-associated oral cancers and treatment strategies. J Dent Res. 2014;93:29S-36S. 10.1177/0022034514527969. 24663683 10.1177/0022034514527969 PMC4107541

[7] Jeon S, Lambert PF. Integration of human papillomavirus type 16 DNA into the human genome leads to increased stability of E6 and E7 mRNAs: implications for cervical carcinogenesis. Proc Natl Acad Sci. 1995;92:1654–8. 10.1073/pnas.92.5.1654. 7878034 10.1073/pnas.92.5.1654 PMC42578

[8] McBride AA, Warburton A. The role of integration in oncogenic progression of HPV-associated cancers. PLOS Pathog. 2017;13: e1006211. 10.1371/journal.ppat.1006211. 28384274 10.1371/journal.ppat.1006211 PMC5383336

[9] Parfenov M, Pedamallu CS, Gehlenborg N, Freeman SS, Danilova L, Bristow CA, et al. Characterization of HPV and host genome interactions in primary head and neck cancers. Proc Natl Acad Sci. 2014;111:15544–9. 10.1073/pnas.1416074111. 25313082 10.1073/pnas.1416074111 PMC4217452

[10] The Cancer Genome Atlas Research Network. Integrated genomic and molecular characterization of cervical cancer. Nature. 2017;543:378–84. 10.1038/nature21386. 28112728 10.1038/nature21386 PMC5354998

[11] Vinokurova S, Wentzensen N, Kraus I, Klaes R, Driesch C, Melsheimer P, et al. Type-dependent integration frequency of human papillomavirus genomes in cervical lesions. Cancer Res. 2008;68:307–13. 10.1158/0008-5472.CAN-07-2754. 18172324 10.1158/0008-5472.CAN-07-2754

[12] Huang J, Qian Z, Gong Y, Wang Y, Guan Y, Han Y, et al. Comprehensive genomic variation profiling of cervical intraepithelial neoplasia and cervical cancer identifies potential targets for cervical cancer early warning. J Med Genet. 2019;56:186–94. 10.1136/jmedgenet-2018-105745. 30567904 10.1136/jmedgenet-2018-105745 PMC6581088

[13] Hu T, Li K, He L, Huang F, Yang F, Chen S, et al. Testing for viral DNA integration among HPV-positive women to detect cervical precancer: an observational cohort study. BJOG Int J Obstet Gynaecol. 2024;131:309–18. 10.1111/1471-0528.17597. 10.1111/1471-0528.17597 37408516

[14] Pan C, Issaeva N, Yarbrough WG. HPV-driven oropharyngeal cancer: current knowledge of molecular biology and mechanisms of carcinogenesis. Cancers Head Neck. 2018;3:12. 10.1186/s41199-018-0039-3. 31093365 10.1186/s41199-018-0039-3 PMC6460765

[15] Menezes FDS, Fernandes GA, Antunes JLF, Villa LL, Toporcov TN. Global incidence trends in head and neck cancer for HPV-related and -unrelated subsites: a systematic review of population-based studies. Oral Oncol. 2021;115: 105177. 10.1016/j.oraloncology.2020.105177. 33561611 10.1016/j.oraloncology.2020.105177

[16] Chaturvedi AK, Graubard BI, Broutian T, Pickard RKL, Tong Z-Y, Xiao W, et al. Effect of prophylactic human papillomavirus (HPV) vaccination on oral HPV infections among young adults in the United States. J Clin Oncol. 2018;36:262–7. 10.1200/JCO.2017.75.0141. 29182497 10.1200/JCO.2017.75.0141 PMC5773841

[17] Pingali C, Yankey D, Elam-Evans LD, Markowitz LE, Williams CL, Fredua B, et al. National, regional, state, and selected local area vaccination coverage among adolescents aged 13–17 Years—United States, 2020. MMWR Morb Mortal Wkly Rep. 2021;70:1183–90. 10.15585/mmwr.mm7035a1. 34473682 10.15585/mmwr.mm7035a1 PMC8422873

[18] Athanasiou A, Bowden S, Paraskevaidi M, Fotopoulou C, Martin-Hirsch P, Paraskevaidis E, et al. HPV vaccination and cancer prevention. Best Pract Res Clin Obstet Gynaecol. 2020;65:109–24. 10.1016/j.bpobgyn.2020.02.009. 32284298 10.1016/j.bpobgyn.2020.02.009

[19] Zhang Y, Fakhry C, D’Souza G. Projected association of human papillomavirus vaccination with oropharynx cancer incidence in the US, 2020–2045. JAMA Oncol. 2021;7: e212907. 10.1001/jamaoncol.2021.2907. 34473210 10.1001/jamaoncol.2021.2907 PMC8414358

[20] Ruffin AT, Li H, Vujanovic L, Zandberg DP, Ferris RL, Bruno TC. Improving head and neck cancer therapies by immunomodulation of the tumour microenvironment. Nat Rev Cancer. 2023;23:173–88. 10.1038/s41568-022-00531-9. 36456755 10.1038/s41568-022-00531-9 PMC9992112

[21] Johnson DE, Burtness B, Leemans CR, Lui VWY, Bauman JE, Grandis JR. Head and neck squamous cell carcinoma. Nat Rev Dis Primer. 2020;6:92. 10.1038/s41572-020-00224-3. 10.1038/s41572-020-00224-3 PMC7944998 33243986

[22] Rathod S, Livergant J, Klein J, Witterick I, Ringash J. A systematic review of quality of life in head and neck cancer treated with surgery with or without adjuvant treatment. Oral Oncol. 2015;51:888–900. 10.1016/j.oraloncology.2015.07.002. 26209066 10.1016/j.oraloncology.2015.07.002

[23] Spurgeon ME, Lambert PF. Mus musculus papillomavirus 1: a new frontier in animal models of papillomavirus pathogenesis. J Virol. 2020;94:e00002-20. 10.1128/JVI.00002-20. 32051276 10.1128/JVI.00002-20 PMC7163119

[24] Lee TW, Lai A, Harms JK, Singleton DC, Dickson BD, Macann AMJ, et al. Patient-derived xenograft and organoid models for precision medicine targeting of the tumour microenvironment in head and neck cancer. Cancers. 2020;12:3743. 10.3390/cancers12123743. 33322840 10.3390/cancers12123743 PMC7763264

[25] Bouabe H, Okkenhaug K. Gene targeting in mice: a review. In: Bailer SM, Lieber D, editors. Virus-host interact, vol. 1064. Totowa. Humana Press: NJ; 2013. p. 315–36. 10.1007/978-1-62703-601-6_23. 10.1007/978-1-62703-601-6_23 PMC4524968 23996268

[26] Song S, Pitot HC, Lambert PF. The human papillomavirus type 16 E6 gene alone is sufficient to induce carcinomas in transgenic animals. J Virol. 1999;73:5887–93. 10.1128/JVI.73.7.5887-5893.1999. 10364340 10.1128/jvi.73.7.5887-5893.1999 PMC112649

[27] Herber R, Liem A, Pitot H, Lambert PF. Squamous epithelial hyperplasia and carcinoma in mice transgenic for the human papillomavirus type 16 E7 oncogene. J Virol. 1996;70:1873–81. 10.1128/jvi.70.3.1873-1881.1996. 8627712 10.1128/jvi.70.3.1873-1881.1996 PMC190015

[28] Liu Y, Maya S, Ploss A. Animal models of hepatitis b virus infection-success, challenges, and future directions. Viruses. 2021;13:777. 10.3390/v13050777. 33924793 10.3390/v13050777 PMC8146732

[29] Trimble CL, Frazer IH. Development of therapeutic HPV vaccines. Lancet Oncol. 2009;10:975–80. 10.1016/S1470-2045(09)70227-X. 19796749 10.1016/S1470-2045(09)70227-X PMC3090037

[30] Lee JH, Yi SMP, Anderson ME, Berger KL, Welsh MJ, Klingelhutz AJ, et al. Propagation of infectious human papillomavirus type 16 by using an adenovirus and Cre/LoxP mechanism. Proc Natl Acad Sci. 2004;101:2094–9. 10.1073/pnas.0308615100. 14769917 10.1073/pnas.0308615100 PMC357057

[31] Zhong R, Pytynia M, Pelizzari C, Spiotto M. Bioluminescent imaging of HPV-positive oral tumor growth and its response to image-guided radiotherapy. Cancer Res. 2014;74:2073–81. 10.1158/0008-5472.CAN-13-2993. 24525739 10.1158/0008-5472.CAN-13-2993 PMC4662542

[32] Carper MB, Troutman S, Wagner BL, Byrd KM, Selitsky SR, Parag-Sharma K, et al. An immunocompetent mouse model of HPV16(+) head and neck squamous cell carcinoma. Cell Rep. 2019;29:1660-1674.e7. 10.1016/j.celrep.2019.10.005. 31693903 10.1016/j.celrep.2019.10.005 PMC6870917

[33] Soriano P. Generalized lacZ expression with the ROSA26 Cre reporter strain. Nat Genet. 1999;21:70–1. 10.1038/5007. 9916792 10.1038/5007

[34] Lin Y-H, Yang M-C, Tseng S-H, Jiang R, Yang A, Farmer E, et al. Integration of oncogenes via sleeping beauty as a mouse model of HPV16+ oral tumors and immunologic control. Cancer Immunol Res. 2018;6:305–19. 10.1158/2326-6066.CIR-16-0358. 29362220 10.1158/2326-6066.CIR-16-0358 PMC6056342

[35] Thorland EC, Myers SL, Gostout BS, Smith DI. Common fragile sites are preferential targets for HPV16 integrations in cervical tumors. Oncogene. 2003;22:1225–37. 10.1038/sj.onc.1206170. 12606949 10.1038/sj.onc.1206170

[36] Fan J, Fu Y, Peng W, Li X, Shen Y, Guo E, et al. Multi-omics characterization of silent and productive HPV integration in cervical cancer. Cell Genom. 2023;3: 100211. 10.1016/j.xgen.2022.100211. 36777180 10.1016/j.xgen.2022.100211 PMC9903858

[37] Hu Z, Zhu D, Wang W, Li W, Jia W, Zeng X, et al. Genome-wide profiling of HPV integration in cervical cancer identifies clustered genomic hot spots and a potential microhomology-mediated integration mechanism. Nat Genet. 2015;47:158–63. 10.1038/ng.3178. 25581428 10.1038/ng.3178

[38] Bodelon C, Untereiner ME, Machiela MJ, Vinokurova S, Wentzensen N. Genomic characterization of viral integration sites in HPV-related cancers. Int J Cancer. 2016;139:2001–11. 10.1002/ijc.30243. 27343048 10.1002/ijc.30243 PMC6749823

[39] Network CGA. Comprehensive genomic characterization of head and neck squamous cell carcinomas. Nature. 2015;517:576–82. 10.1038/nature14129. 25631445 10.1038/nature14129 PMC4311405

[40] Ziegert C, Wentzensen N, Vinokurova S, Kisseljov F, Einenkel J, Hoeckel M, et al. A comprehensive analysis of HPV integration loci in anogenital lesions combining transcript and genome-based amplification techniques. Oncogene. 2003;22:3977–84. 10.1038/sj.onc.1206629. 12813471 10.1038/sj.onc.1206629

[41] Warburton A, Markowitz TE, Katz JP, Pipas JM, McBride AA. Recurrent integration of human papillomavirus genomes at transcriptional regulatory hubs. Npj Genom Med. 2021;6:101. 10.1038/s41525-021-00264-y. 34848725 10.1038/s41525-021-00264-y PMC8632991

[42] Mou H, Ozata DM, Smith JL, Sheel A, Kwan S-Y, Hough S, et al. CRISPR-SONIC: targeted somatic oncogene knock-in enables rapid in vivo cancer modeling. Genome Med. 2019;11:21. 10.1186/s13073-019-0627-9. 30987660 10.1186/s13073-019-0627-9 PMC6466773

[43] He X, Tan C, Wang F, Wang Y, Zhou R, Cui D, et al. Knock-in of large reporter genes in human cells via CRISPR/Cas9-induced homology-dependent and independent DNA repair. Nucleic Acids Res. 2016;44:e85–e85. 10.1093/nar/gkw064. 26850641 10.1093/nar/gkw064 PMC4872082

[44] Wood ZC, Bain CJ, Smith DD, Whiteman DC, Antonsson A. Oral human papillomavirus infection incidence and clearance: a systematic review of the literature. J Gen Virol. 2017;98:519–26. 10.1099/jgv.0.000727. 28150575 10.1099/jgv.0.000727

[45] Sun Q, Wang L, Zhang C, Hong Z, Han Z. Cervical cancer heterogeneity: a constant battle against viruses and drugs. Biomark Res. 2022;10:85. 10.1186/s40364-022-00428-7. 36397138 10.1186/s40364-022-00428-7 PMC9670454

[46] Vogelstein B, Kinzler KW. The multistep nature of cancer. Trends Genet. 1993;9:138–41. 10.1016/0168-9525(93)90209-Z. 8516849 10.1016/0168-9525(93)90209-z

[47] Fernández-Mateos J, Pérez-García J, Seijas-Tamayo R, Mesía R, Rubió-Casadevall J, García-Girón C, et al. Oncogenic driver mutations predict outcome in a cohort of head and neck squamous cell carcinoma (HNSCC) patients within a clinical trial. Sci Rep. 2020;10:16634. 10.1038/s41598-020-72927-2. 33024167 10.1038/s41598-020-72927-2 PMC7539152

[48] Akbari Dilmaghani N, Safaroghli-Azar A, Pourbagheri-Sigaroodi A, Bashash D. The PI3K/Akt/mTORC signaling axis in head and neck squamous cell carcinoma: Possibilities for therapeutic interventions either as single agents or in combination with conventional therapies. IUBMB Life. 2021;73:618–42. 10.1002/iub.2446. 33476088 10.1002/iub.2446

[49] Aguayo F, Perez-Dominguez F, Osorio JC, Oliva C, Calaf GM. PI3K/AKT/mTOR signaling pathway in HPV-driven head and neck carcinogenesis: therapeutic implications. Biology. 2023;12:672. 10.3390/biology12050672. 37237486 10.3390/biology12050672 PMC10215516

[50] Chernock RD. Morphologic features of conventional squamous cell carcinoma of the oropharynx: ‘keratinizing’ and ‘nonkeratinizing’ histologic types as the basis for a consistent classification system. Head Neck Pathol. 2012;6:41–7. 10.1007/s12105-012-0373-4. 10.1007/s12105-012-0373-4 PMC3394167 22782222

[51] Krüger M, Pabst AM, Walter C, Sagheb K, Günther C, Blatt S, et al. The prevalence of human papilloma virus (HPV) infections in oral squamous cell carcinomas: a retrospective analysis of 88 patients and literature overview. J Cranio-Maxillofac Surg. 2014;42:1506–14. 10.1016/j.jcms.2014.04.022. 10.1016/j.jcms.2014.04.022 24947612

[52] Majchrzak E, Szybiak B, Wegner A, Pienkowski P, Pazdrowski J, Luczewski L, et al. Oral cavity and oropharyngeal squamous cell carcinoma in young adults: a review of the literature. Radiol Oncol. 2014;48:1–10. 10.2478/raon-2013-0057. 24587773 10.2478/raon-2013-0057 PMC3908841

[53] Henkle TR, Lam B, Kung YJ, Lin J, Tseng S-H, Ferrall L, et al. Development of a novel mouse model of spontaneous high-risk HPVE6/E7–expressing carcinoma in the cervicovaginal tract. Cancer Res. 2021;81:4560–9. 10.1158/0008-5472.CAN-21-0399. 34215618 10.1158/0008-5472.CAN-21-0399 PMC8416934

[54] Hung C-F, Cheng W-F, Hsu K-F, Chai C-Y, He L, Ling M, et al. Cancer immunotherapy using a DNA vaccine encoding the translocation domain of a bacterial toxin linked to a tumor antigen1. Cancer Res. 2001;61:3698–703. 11325841

[55] Carlson CM, Frandsen JL, Kirchhof N, McIvor RS, Largaespada DA. Somatic integration of an oncogene-harboring *Sleeping Beauty* transposon models liver tumor development in the mouse. Proc Natl Acad Sci. 2005;102:17059–64. 10.1073/pnas.0502974102. 16286660 10.1073/pnas.0502974102 PMC1287966

[56] Wiesner SM, Decker SA, Larson JD, Ericson K, Forster C, Gallardo JL, et al. *De novo* induction of genetically engineered brain tumors in mice using plasmid DNA. Cancer Res. 2009;69:431–9. 10.1158/0008-5472.CAN-08-1800. 19147555 10.1158/0008-5472.CAN-08-1800 PMC2701484

[57] Mátés L, Chuah MKL, Belay E, Jerchow B, Manoj N, Acosta-Sanchez A, et al. Molecular evolution of a novel hyperactive Sleeping Beauty transposase enables robust stable gene transfer in vertebrates. Nat Genet. 2009;41:753–61. 10.1038/ng.343. 19412179 10.1038/ng.343

[58] Lin KY, Guarnieri FG, Staveley-O’Carroll KF, Levitsky HI, August JT, Pardoll DM, et al. Treatment of established tumors with a novel vaccine that enhances major histocompatibility class II presentation of tumor antigen. Cancer Res. 1996;56:21–6. 8548765

[59] Cheng W-F, Hung C-F, Chai C-Y, Hsu K-F, He L, Ling M, et al. Tumor-specific immunity and antiangiogenesis generated by a DNA vaccine encoding calreticulin linked to a tumor antigen. J Clin Invest. 2001;108:669–78. 10.1172/JCI200112346. 11544272 10.1172/JCI12346 PMC209378

[60] Kim JW, Hung C-F, Juang J, He L, Kim TW, Armstrong DK, et al. Comparison of HPV DNA vaccines employing intracellular targeting strategies. Gene Ther. 2004;11:1011–8. 10.1038/sj.gt.3302252. 14985791 10.1038/sj.gt.3302252

[61] Holman PO, Walsh ER, Jameson SC. Characterizing the impact of CD8 antibodies on class I MHC multimer binding. J Immunol. 2005;174:3986–91. 10.4049/jimmunol.174.7.3986. 15778355 10.4049/jimmunol.174.7.3986

[62] Clement M, Ladell K, Ekeruche-Makinde J, Miles JJ, Edwards ESJ, Dolton G, et al. Anti-CD8 antibodies can trigger CD8+ T cell effector function in the absence of TCR engagement and improve peptide–MHCI tetramer staining. J Immunol. 2011;187:654–63. 10.4049/jimmunol.1003941. 21677135 10.4049/jimmunol.1003941 PMC3145095

[63] Peng S, Song L, Knoff J, Wang JW, Chang Y-N, Hannaman D, et al. Control of HPV-associated tumors by innovative therapeutic HPV DNA vaccine in the absence of CD4+ T cells. Cell Biosci. 2014;4:11. 10.1186/2045-3701-4-11. 24594273 10.1186/2045-3701-4-11 PMC4015858

[64] Jagadeeshan S, Novoplansky OZ, Cohen O, Kurth I, Hess J, Rosenberg AJ, et al. New insights into RAS in head and neck cancer. Biochim Biophys Acta BBA Rev Cancer. 2023;1878: 188963. 10.1016/j.bbcan.2023.188963. 10.1016/j.bbcan.2023.188963 PMC11815531 37619805

[65] Novoplansky O, Jagadeeshan S, Regev O, Menashe I, Elkabets M. Worldwide prevalence and clinical characteristics of RAS mutations in head and neck cancer: a systematic review and meta-analysis. Front Oncol. 2022;12: 838911. 10.3389/fonc.2022.838911. 35600380 10.3389/fonc.2022.838911 PMC9121358

[66] Wang X, Xu Z, Tian Z, Zhang X, Xu D, Li Q, et al. The EF-1α promoter maintains high-level transgene expression from episomal vectors in transfected CHO-K1 cells. J Cell Mol Med. 2017;21:3044–54. 10.1111/jcmm.13216. 28557288 10.1111/jcmm.13216 PMC5661254

[67] Baklaushev VP, Kilpeläinen A, Petkov S, Abakumov MA, Grinenko NF, Yusubalieva GM, et al. Luciferase expression allows bioluminescence imaging but imposes limitations on the orthotopic mouse (4T1) model of breast cancer. Sci Rep. 2017;7:7715. 10.1038/s41598-017-07851-z. 28798322 10.1038/s41598-017-07851-z PMC5552689

[68] Barrett DM, Seif AE, Carpenito C, Strong EP, June CH, Grupp SA, et al. Bioluminescent tracking of human and mouse acute lymphoblastic leukemia reveals potent immunogenicity of luciferase in some preclinical models of leukemia. Blood. 2010;116:2140–2140. 10.1182/blood.V116.21.2140.2140.

[69] Day C-P, Carter J, Ohler ZW, Bonomi C, El Meskini R, Martin P, et al. “Glowing head” mice: a genetic tool enabling reliable preclinical image-based evaluation of cancers in immunocompetent allografts. PLoS ONE. 2014;9: e109956. 10.1371/journal.pone.0109956. 25369133 10.1371/journal.pone.0109956 PMC4219677

[70] Trotter TN, Wilson A, McBane J, Dagotto CE, Yang X-Y, Wei J-P, et al. Overcoming Xenoantigen immunity to enable cellular tracking and gene regulation with immune-competent “Noglow” mice. Cancer Res Commun. 2024;4:1050–62. 10.1158/2767-9764.CRC-24-0062. 38592453 10.1158/2767-9764.CRC-24-0062 PMC11003454

[71] Govan VA. Strategies for human papillomavirus therapeutic vaccines and other therapies based on the E6 and E7 oncogenes. Ann N Y Acad Sci. 2005;1056:328–43. 10.1196/annals.1352.016. 16387699 10.1196/annals.1352.016

[72] Rotnáglová E, Tachezy R, Saláková M, Procházka B, Košl’abová E, Veselá E, et al. HPV involvement in tonsillar cancer: prognostic significance and clinically relevant markers. Int J Cancer. 2011;129:101–10. 10.1002/ijc.25889. 21190188 10.1002/ijc.25889

[73] Kofler B, Laban S, Busch CJ, Lörincz B, Knecht R. New treatment strategies for HPV-positive head and neck cancer. Eur Arch Otorhinolaryngol. 2014;271:1861–7. 10.1007/s00405-013-2603-0. 23934317 10.1007/s00405-013-2603-0

[74] Yuan Y, Cai X, Shen F, Ma F. HPV post-infection microenvironment and cervical cancer. Cancer Lett. 2021;497:243–54. 10.1016/j.canlet.2020.10.034. 33122098 10.1016/j.canlet.2020.10.034

[75] Lo Cigno I, Calati F, Albertini S, Gariglio M. Subversion of host innate immunity by human papillomavirus oncoproteins. Pathogens. 2020;9:292. 10.3390/pathogens9040292. 32316236 10.3390/pathogens9040292 PMC7238203

[76] Scott ML, Woodby BL, Ulicny J, Raikhy G, Orr AW, Songock WK, et al. Human papillomavirus 16 E5 inhibits interferon signaling and supports episomal viral maintenance. J Virol. 2020;94:e01582-e1619. 10.1128/JVI.01582-19. 31666385 10.1128/JVI.01582-19 PMC6955282

[77] Frazer IH. Interaction of human papillomaviruses with the host immune system: a well evolved relationship. Virology. 2009;384:410–4. 10.1016/j.virol.2008.10.004. 18986661 10.1016/j.virol.2008.10.004

[78] Woodby B, Scott M, Bodily J. The interaction between human papillomaviruses and the stromal microenvironment. Prog Mol Biol Transl Sci. 2016;144:169–238. 10.1016/bs.pmbts.2016.09.003. 27865458 10.1016/bs.pmbts.2016.09.003 PMC5727914

[79] Looker KJ, Rönn MM, Brock PM, Brisson M, Drolet M, Mayaud P, et al. Evidence of synergistic relationships between HIV and Human Papillomavirus ( HPV ): systematic reviews and meta-analyses of longitudinal studies of HPV acquisition and clearance by HIV status, and of HIV acquisition by HPV status. J Int AIDS Soc. 2018;21: e25110. 10.1002/jia2.25110. 29873885 10.1002/jia2.25110 PMC5989783

[80] Nailescu C, Ermel AC, Shew ML. Human papillomavirus-related cancer risk for solid organ transplant recipients during adult life and early prevention strategies during childhood and adolescence. Pediatr Transplant. 2022;26: e14341. 10.1111/petr.14341. 35808949 10.1111/petr.14341

[81] Madeleine MM, Finch JL, Lynch CF, Goodman MT, Engels EA. HPV-related cancers after solid organ transplantation in the United States. Am J Transplant. 2013;13:3202–9. 10.1111/ajt.12472. 24119294 10.1111/ajt.12472 PMC4049182

[82] Arbeit JM, Münger K, Howley PM, Hanahan D. Progressive squamous epithelial neoplasia in K14-human papillomavirus type 16 transgenic mice. J Virol. 1994;68:4358–68. 10.1128/jvi.68.7.4358-4368.1994. 7515971 10.1128/jvi.68.7.4358-4368.1994 PMC236359

[83] Vassar R, Fuchs E. Transgenic mice provide new insights into the role of TGF-alpha during epidermal development and differentiation. Genes Dev. 1991;5:714–27. 10.1101/gad.5.5.714. 1709129 10.1101/gad.5.5.714

[84] Lim YX, D’Silva NJ. HPV-associated oropharyngeal cancer: in search of surrogate biomarkers for early lesions. Oncogene. 2024;43:543–54. 10.1038/s41388-023-02927-9. 38191674 10.1038/s41388-023-02927-9 PMC10873204

[85] Mahal BA, Catalano PJ, Haddad RI, Hanna GJ, Kass JI, Schoenfeld JD, et al. Incidence and demographic burden of HPV-associated oropharyngeal head and neck cancers in the United States. Cancer Epidemiol Biomarkers Prev. 2019;28:1660–7. 10.1158/1055-9965.EPI-19-0038. 31358520 10.1158/1055-9965.EPI-19-0038

[86] Combes J-D, Franceschi S. Role of human papillomavirus in non-oropharyngeal head and neck cancers. Oral Oncol. 2014;50:370–9. 10.1016/j.oraloncology.2013.11.004. 24331868 10.1016/j.oraloncology.2013.11.004

[87] Montine KS, Treuting PM, Dintzis SM, editors. Comparative anatomy and histology: a mouse, rat and human atlas. 2nd ed. London: Academic Press, an imprint of Elsevier; 2018.

[88] McCarty NS, Graham AE, Studená L, Ledesma-Amaro R. Multiplexed CRISPR technologies for gene editing and transcriptional regulation. Nat Commun. 2020;11:1281. 10.1038/s41467-020-15053-x. 32152313 10.1038/s41467-020-15053-x PMC7062760

[89] Pinatti LM, Walline HM, Carey TE. Human papillomavirus genome integration and head and neck cancer. J Dent Res. 2018;97:691–700. 10.1177/0022034517744213. 29227715 10.1177/0022034517744213 PMC5960877

[90] Akagi K, Li J, Broutian TR, Padilla-Nash H, Xiao W, Jiang B, et al. Genome-wide analysis of HPV integration in human cancers reveals recurrent, focal genomic instability. Genome Res. 2014;24:185–99. 10.1101/gr.164806.113. 24201445 10.1101/gr.164806.113 PMC3912410

[91] Walline HM, Goudsmit CM, McHugh JB, Tang AL, Owen JH, Teh BT, et al. Integration of high-risk human papillomavirus into cellular cancer-related genes in head and neck cancer cell lines. Head Neck. 2017;39:840–52. 10.1002/hed.24729. 28236344 10.1002/hed.24729 PMC5392184

[92] Pinatti LM, Gu W, Wang Y, Elhossiny A, Bhangale AD, Brummel CV, et al. SearcHPV: a novel approach to identify and assemble human papillomavirus–host genomic integration events in cancer. Cancer. 2021;127:3531–40. 10.1002/cncr.33691. 34160069 10.1002/cncr.33691 PMC8454028

[93] Mainguené J, Vacher S, Kamal M, Hamza A, Masliah-Planchon J, Baulande S, et al. Human papilloma virus integration sites and genomic signatures in head and neck squamous cell carcinoma. Mol Oncol. 2022;16:3001–16. 10.1002/1878-0261.13219. 35398964 10.1002/1878-0261.13219 PMC9394244

