# Supplementary material for: Generation of a spontaneous murine HPV + oral cancer model with site-specific oncogene insertion using CRISPR-SONIC
Source: Cell Biosci. 2025 Jun 18;15:84. doi: 10.1186/s13578-025-01427-5 (PMC12175459; doi:10.1186/s13578-025-01427-5)
Supplement: Supplementary file 1 [file 13578_2025_1427_MOESM1_ESM.pdf]

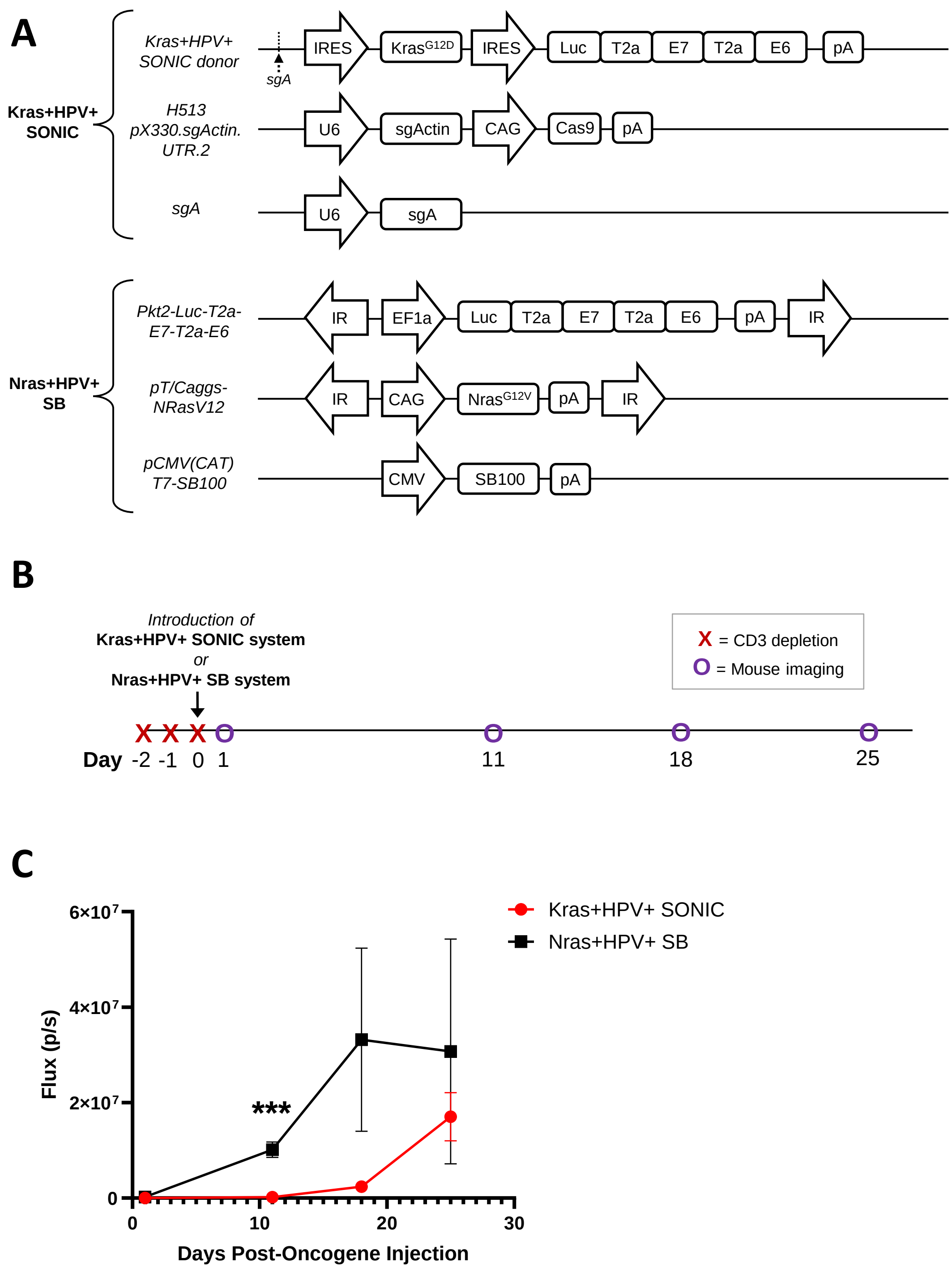

A
Kras+HPV+ SONIC
Kras+HPV+ SONIC donor
sgA
IRES
KrasG12D
IRES
Luc
T2a
E7
T2a
E6
pA
H513 pX330.sgActin. UTR.2
U6
sgActin
CAG
Cas9
pA
sgA
U6
sgA
Nras+HPV+ SB
Pkt2-Luc-T2a-E7-T2a-E6
IR
EF1a
Luc
T2a
E7
T2a
E6
pA
IR
pT/Caggs-NRasV12
IR
CAG
NrasG12V
pA
IR
pCMV(CAT) T7-SB100
CMV
SB100
pA
B
Introduction of
Kras+HPV+ SONIC system
or
Nras+HPV+ SB system
X = CD3 depletion
O = Mouse imaging
Day -2 -1 0 1 11 18 25
C
Kras+HPV+ SONIC
Nras+HPV+ SB
6×10⁷
4×10⁷
2×10⁷
0
Flux (p/s)
***
0
10
20
30
Days Post-Oncogene Injection

Figure S1. CRISPR-SONIC system generates lower expression of genes from donor vector *in vitro* and *in vivo* compared to SB system. A) Schematic diagram of plasmids in the Kras+HPV+ SONIC system and Nras+HPV+ SB system used for tumor generation in two separate groups of C57BL/6 mice (n=5). All plasmids were administered via submucosal injection followed by electroporation. Tumor growth by bioluminescence signal was quantified by imaging on the IVIS Spectrum after intraperitoneal injection of luciferin. **B)** Schedule of CD3 depletion and bioluminescence imaging of buccal tumors. Kras+HPV+ SONIC or Nras+HPV+ SB was introduced in the buccal region of each group of mice through submucosal injection and electroporation on Day 0, as indicated. **C)** Quantified bioluminescence kinetics of buccal tumors in mice with an initial 3-day anti-CD3 administration prior to administration of Kras+HPV+ SONIC system or Nras+HPV+ SB system. *Data represent the means ±SEM. Unpaired two-tailed t-test analysis was conducted. In all cases, p≤0.05 was considered statistically significant. (*p < 0.05, **p < 0.01, ***p < 0.001, ****p<0.0001 vs control)*

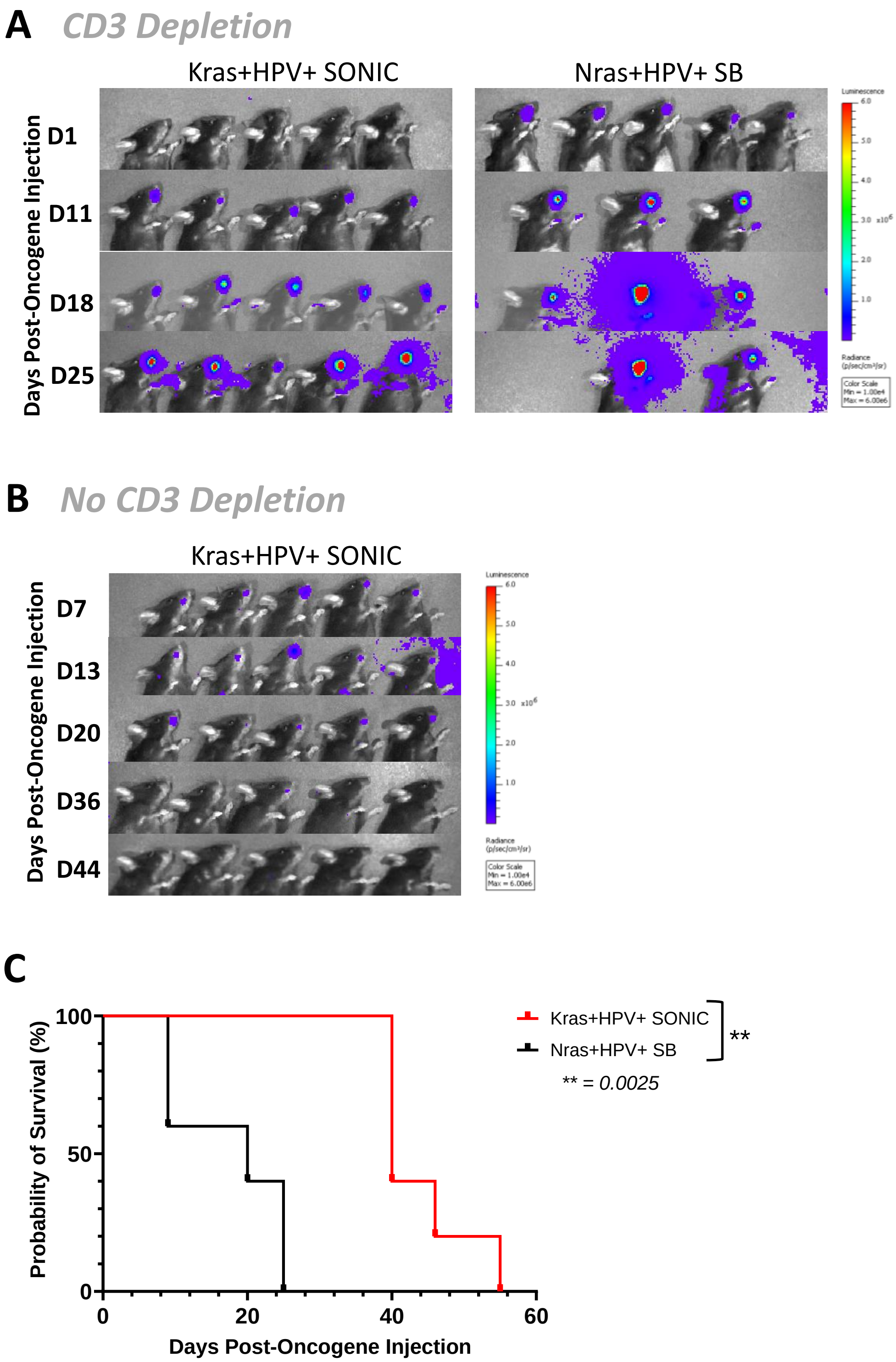

A
CD3 Depletion
Kras+HPV+ SONIC
Nras+HPV+ SB
Days Post-Oncogene Injection
D1
D11
D18
D25
Luminescence
6.0
5.0
4.0
3.0 x10^6
2.0
1.0
Radiance (p/sec/cm²/sr)
Color Scale Min = 1.00e4 Max = 6.00e6
B
No CD3 Depletion
Kras+HPV+ SONIC
Days Post-Oncogene Injection
D7
D13
D20
D36
D44
Luminescence
6.0
5.0
4.0
3.0 x10^6
2.0
1.0
Radiance (p/sec/cm²/sr)
Color Scale Min = 1.00e4 Max = 6.00e6
C
Probability of Survival (%)
100
50
0
0
20
40
60
Days Post-Oncogene Injection
Kras+HPV+ SONIC
Nras+HPV+ SB
**
** = 0.0025

Figure S2. CRISPR-SONIC-induced buccal tumors in C57BL/6 mice require transient immunosuppression and result in reduced tumor-related mortality compared to SB system. A) Bioluminescence imaging of buccal tumor growth in C57BL/6 mice administered Kras+HPV+ SONIC or Nras+HPV+ SB system with an initial 3-day anti-CD3 administration prior to oncogene injection. **B)** Bioluminescence imaging of buccal tumor growth in C57BL/6 mice administered Kras+HPV+ SONIC system without prior immune depletion. **C)** Kaplan-Meier survival plot of CD3-depleted C57BL/6 mice administered Kras+HPV+ SONIC or Nras+HPV+ SB system. *The log-rank test was used to compare survival outcomes between groups. A $p \leq 0.05$ was considered statistically significant. (*$p < 0.05$, **$p < 0.01$, ***$p < 0.001$, ****$p<0.0001$ vs control).*

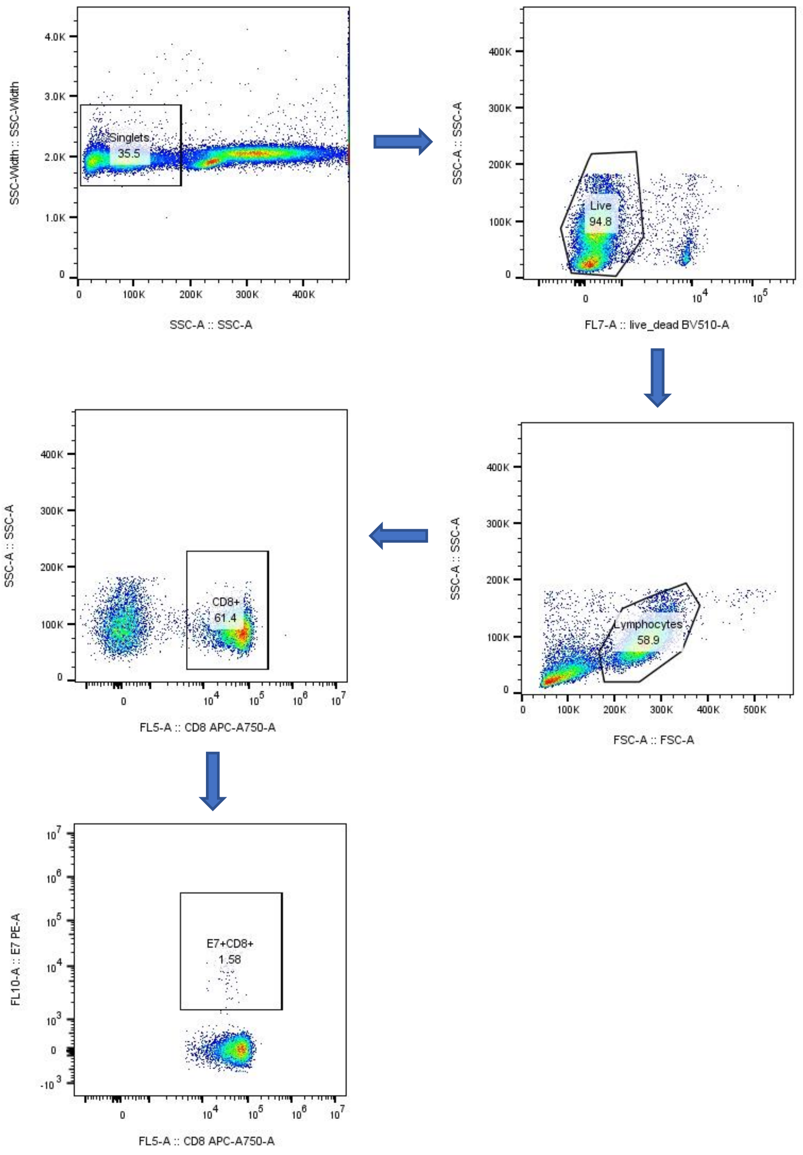

Figure S3. E7-specific CD8 T cell population gating strategy for flow cytometry analysis experiments. Single cells were first gated based on SSC-W and SSC-A, and live cells were gated based on SSC-A and Zombie Aqua Live/Dead stain. Lymphocytes were then gated based on SSC-A and FSC-A, and CD8+ cells were gated based on CD8 expression. The E7+CD8+ subpopulation was then further gated based on E7 expression.

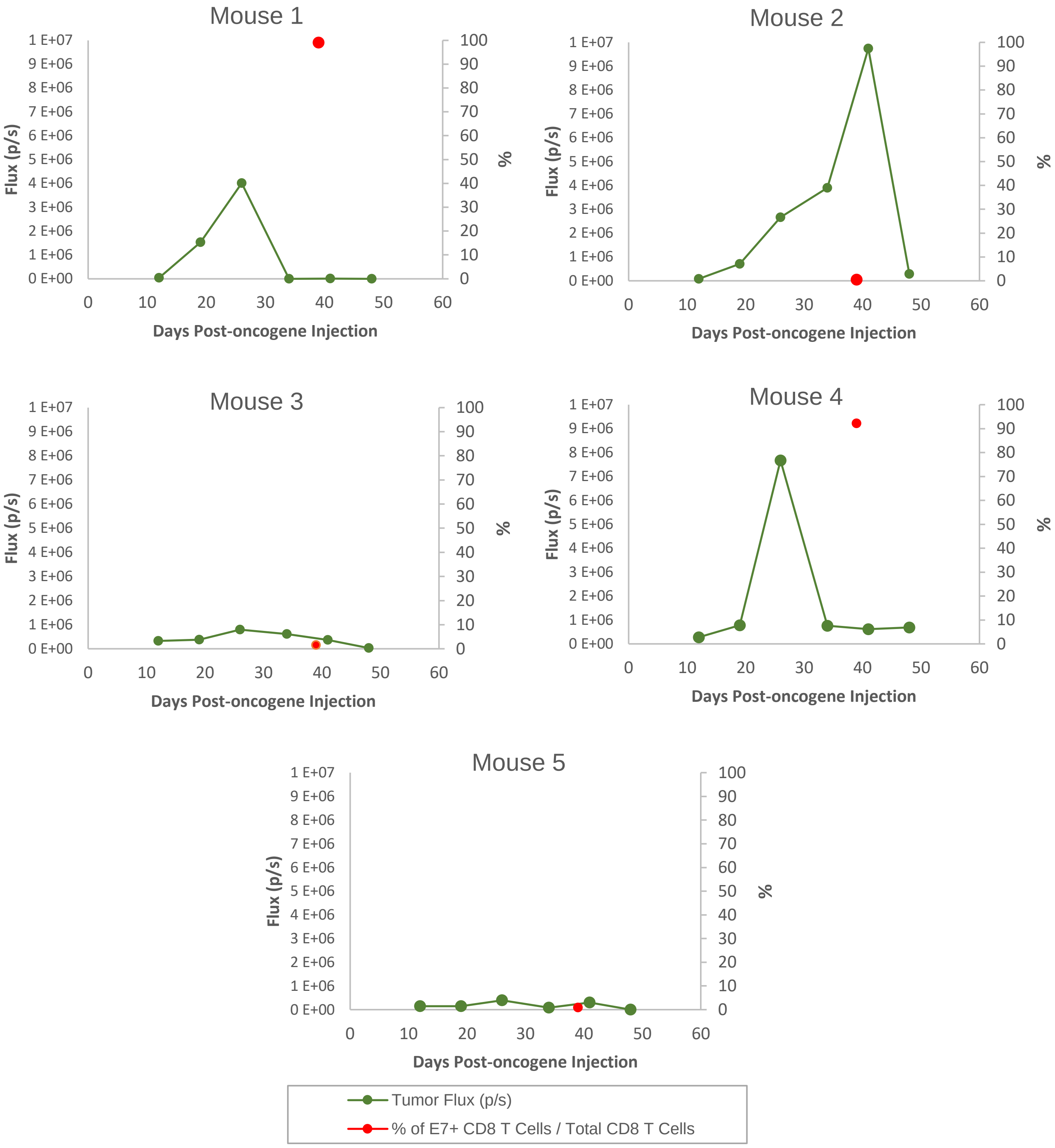
Mouse 1
Mouse 2
Mouse 3
Mouse 4
Mouse 5
Flux (p/s)
%
1 E+07
9 E+06
8 E+06
7 E+06
6 E+06
5 E+06
4 E+06
3 E+06
2 E+06
1 E+06
0 E+00
100
90
80
70
60
50
40
30
20
10
0
0
10
20
30
40
50
60
Days Post-oncogene Injection
Tumor Flux (p/s)
% of E7+ CD8 T Cells / Total CD8 T Cells

Figure S4. Individual tumor growth kinetics as measured via bioluminescence imaging in CD4-depleted C57BL/6 mice receiving therapeutic vaccination with pNGVL4a-CRT/E7(detox) DNA vaccine. Bioluminescence-based tumor flux kinetics (green) were measured over time in individual buccal tumor-bearing mice (n=5) post-oncogene injection. The corresponding percentage of E7-specific CD8 T cells (% of E7+ CD8 T cells out of total CD8 T cells, red) was quantified on day 39 post-oncogene injection. The data show a trend suggestive of an inverse relationship between relative tumor burden and E7-specific CD8 T cell response at the time of measurement.

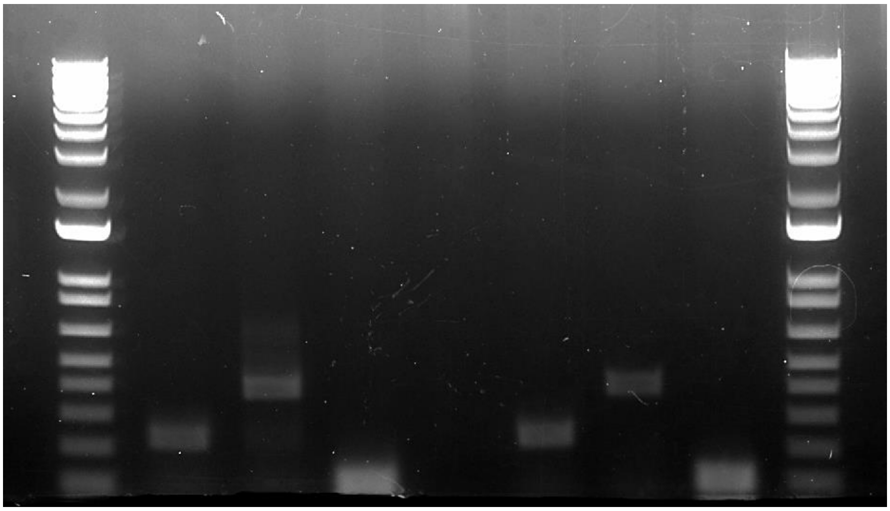

E7 E6 Actb E7 E6 Actb

Sample #1 **Sample #2**

Figure S5. HPV16 E6 and E7 are expressed in tumors generated by the SONIC system. RNA was extracted from tumors of two NSG mice administered with the HPV+ SONIC system with *AKT* and *c-Myc* oncogene co-delivery, and was reverse transcribed to cDNA. PCR was used to amplify E6 and E7 sequences, and agarose gel electrophoresis (1%) of PCR product was performed for visualization.
